# Efficacy of Low‐Voltage Area Ablation Across Substrate Size in Persistent Atrial Fibrillation: A Post Hoc Analysis of the SUPPRESS‐AF Randomized Trial

**DOI:** 10.1161/JAHA.125.047814

**Published:** 2026-05-25

**Authors:** Masato Okada, Daisuke Sakamoto, Masaharu Masuda, Nobuaki Tanaka, Tetsuya Watanabe, Hitoshi Minamiguchi, Yasuyuki Egami, Takafumi Oka, Tomoko Minamisaka, Masato Kawasaki, Nobuhiko Makino, Takashi Kanda, Yasuhiro Matsuda, Koji Tanaka, Yuko Hirao, Naoko Miyazaki, Kohei Iwasa, Hirota Kida, Shungo Hikoso, Akihiro Sunaga, Tomoharu Dohi, Koichi Inoue, Yohei Sotomi, Yasushi Sakata, OCVC‐Arrhythmia Investigators

**Affiliations:** ^1^ Cardiovascular Center Sakurabashi Watanabe Advanced Healthcare Hospital Osaka Japan; ^2^ Department of Cardiovascular Medicine Osaka University Graduate School of Medicine Suita Japan; ^3^ Cardiovascular Center Kansai Rosai Hospital Amagasaki Japan; ^4^ Division of Cardiology Osaka General Medical Center Osaka Japan; ^5^ Department of Cardiovascular Medicine Yao Municipal Hospital Yao Japan; ^6^ Cardiovascular Division Osaka Keisatsu Hospital Osaka Japan; ^7^ Division of Cardiology Osaka Rosai Hospital Sakai Japan; ^8^ Cardiovascular Medicine Nara Medical University Kashihara Japan; ^9^ Cardiovascular Division National Hospital Organization Osaka National Hospital Osaka Japan

**Keywords:** ablation, atrial fibrillation, low‐voltage areas, recurrence, Atrial Fibrillation

## Abstract

**Background:**

Larger low‐voltage areas (LVAs) in the left atrium are associated with increased arrhythmia recurrence after atrial fibrillation (AF) ablation. The benefit of adjunctive LVA ablation may therefore depend on substrate extent. This study examined the efficacy of LVA ablation across a spectrum of LVA sizes in persistent AF.

**Methods:**

The SUPPRESS‐AF (Efficacy and Safety of Left Atrial Low‐Voltage Area Guided Ablation for Recurrence Prevention Compared to Pulmonary Vein Isolation Alone in Patients With Persistent Atrial Fibrillation) trial screened 1364 patients undergoing initial ablation for persistent AF, of whom 342 with left atrial LVAs (≥5 cm^2^) were randomized to pulmonary vein isolation with or without adjunctive LVA ablation. In the 341 analyzed patients, LVA size was categorized as small (<10 cm^2^, n=106), moderate (≥10 to <20 cm^2^, n=127), or extensive (≥20 cm^2^, n=108). The primary end point was recurrence of AF or atrial tachycardia within 1 year without antiarrhythmic drugs. Treatment effects across LVA sizes were evaluated using a Cox model with restricted cubic splines.

**Results:**

AF/atrial tachycardia recurrence rates were similar between the pulmonary vein isolation+LVA ablation and pulmonary vein isolation‐alone groups in patients with small (38.5% versus 29.6%; *P*=0.28) and moderate LVA sizes (40.6% versus 53.5%; *P*=0.15). However, adjunctive LVA ablation significantly reduced recurrence in patients with extensive LVAs (34.7% versus 57.6%; *P*=0.029; interaction *P*=0.054), driven mainly by lower AF recurrence (18.4% versus 42.4%, *P*=0.008). Spline analysis indicated a greater treatment benefit with increasing LVA size, reaching significance around 20 cm^2^.

**Conclusions:**

The efficacy of adjunctive LVA ablation increased with substrate size, with a significant benefit observed in patients with extensive LVAs. These findings support a substrate size–guided ablation strategy to optimize rhythm outcomes in persistent AF.

**Registration:**

URL: https://www.umin.ac.jp/ctr; Identifier: UMIN000035940.

**Nonstandard Abbreviations and Acronyms**
AT: atrial tachycardia
LVA: low‐voltage area
LVA‐ABL: low‐voltage area ablation
PVI: pulmonary vein isolation

**Clinical Perspective**

**What Is New?**

- This post hoc analysis of the SUPPRESS‐AF (Efficacy and Safety of Left Atrial Low‐Voltage Area Guided Ablation for Recurrence Prevention Compared to Pulmonary Vein Isolation Alone in Patients With Persistent Atrial Fibrillation) trial shows that the efficacy of adjunctive low‐voltage area (LVA) ablation varies according to left atrial substrate size in patients with persistent atrial fibrillation.
- Adjunctive LVA ablation reduces 12‐month atrial fibrillation or atrial tachycardia recurrence mainly in patients with extensive LVAs (≥20 cm^2^), and this difference is primarily driven by a reduction in atrial fibrillation recurrence rather than atrial tachycardia recurrence.

**What Are the Clinical Implications?**

- Assessing LVA size may help individualize ablation strategies by avoiding unnecessary adjunctive ablation in patients with limited LVAs and by considering additional LVA ablation in those with extensive LVAs, while emphasizing procedural safety.

Pulmonary vein isolation (PVI) is the cornerstone of catheter ablation for persistent atrial fibrillation (AF); however, its efficacy is limited, with substantial rates of arrhythmia recurrence.^1^, ^2^ Low‐voltage areas (LVAs) in the left atrium reflect myocardial degeneration and represent arrhythmogenic substrates that contribute to AF persistence and recurrence.^3^, ^4^, ^5^ Consequently, adjunctive substrate modification targeting LVAs has been proposed as a strategy to improve rhythm outcomes in patients with persistent AF.^6^, ^7^, ^8^, ^9^

The SUPPRESS‐AF (Efficacy and Safety of Left Atrial Low‐Voltage Area Guided Ablation for Recurrence Prevention Compared to Pulmonary Vein Isolation Alone in Patients With Persistent Atrial Fibrillation) randomized trial evaluated the efficacy of adjunctive LVA ablation in patients with persistent AF and LVAs ≥5 cm.^2^ ^10^, ^11^ Although the overall trial did not demonstrate a statistically significant reduction in atrial arrhythmia recurrence at 1 year with LVA ablation compared with PVI alone, a prespecified subgroup analysis suggested a potential benefit in patients with larger LVAs (≥20 cm^2^). This raised the possibility that the benefit of LVA ablation may be limited in patients with relatively small LVAs, whereas those with more extensive substrate may derive greater benefit due to their higher baseline arrhythmia risk.

In this context, the present post hoc analysis of the SUPPRESS‐AF trial sought to quantify the size‐dependent efficacy of adjunctive LVA ablation using both categorical and continuous analyses and to explore a potential threshold beyond which the procedure confers clinical benefit.

## METHODS

The data that support the findings of this study are available from the corresponding author upon reasonable request.

### Study Design

The SUPPRESS‐AF trial (UMIN000035940) was a prospective, investigator‐initiated, multicenter, randomized controlled trial conducted by the Osaka Cardiovascular Conference Arrhythmia Investigators. The original trial evaluated the efficacy of adjunctive LVA ablation in patients with persistent AF and left atrial LVAs. Patients were randomly assigned intraprocedurally in a 1:1 ratio to undergo either PVI‐alone or PVI followed by left atrial LVA ablation (PVI+LVA‐ABL). One‐year rhythm outcomes were compared between the groups. All patients provided written informed consent, and the study protocol was approved by the institutional review boards of all participating hospitals and conducted in accordance with the Declaration of Helsinki.

### Patient Selection

Patients with persistent AF scheduled for initial ablation were prospectively screened. Key exclusion criteria included left atrial diameter ≥55 mm, history of cardiac surgery, valvular AF, hemodialysis, recent stroke, treatable causes of AF, or other factors deemed unsuitable by the attending physicians. Valvular AF was defined as AF in the presence of moderate‐to‐severe mitral stenosis or a mechanical prosthetic heart valve.^12^

Of 1364 eligible patients, 1347 underwent PVI followed by left atrial voltage mapping with the CARTO3 mapping system (Biosense Webster, Irvine, CA, USA). LVAs were defined as regions with bipolar peak‐to‐peak voltage <0.50 mV. Among the screened patients, 343 (25.5%) demonstrated LVAs covering ≥5 cm^2^ of the left atrial surface.

After excluding one patient due to an allocation system error, 342 patients were randomized in a 1:1 ratio to either PVI+LVA‐ABL (n=170) or PVI alone (n=172) immediately after voltage mapping. Randomization was computer‐generated through a centralized, concealed process, with each site notified via a secure web‐based system. Excluding 1 additional patient due to a protocol violation, 341 patients were included in the present analysis (Figure S1).

Patients were stratified into 3 groups based on the left atrial LVA size: small (≥5 to <10 cm^2^), moderate (≥10 to <20 cm^2^), and extensive (≥20 cm^2^), consistent with previous studies.^13^, ^14^ Treatment efficacy of PVI+LVA‐ABL versus PVI alone was evaluated within and across these LVA size groups.

### Ablation Procedure

The detailed ablation protocol has been described previously.^10^, ^11^ In brief, ipsilateral circumferential PVI was performed in all patients using the CARTO3 mapping system and an open‐irrigated ablation catheter with a contact force sensor (Thermocool Smarttouch, Biosense Webster). Radiofrequency energy application was guided by the VISITAG Surpoint module (Biosense Webster) with the following target settings: ablation index ≥425 for the anterior wall and ≥375 for the posterior wall, with an interlesion distance of ≤4 mm. The VISITAG module settings were (1) catheter stability range of motion ≤2 mm, (2) stability duration >5 seconds, and (3) contact force ≥5 g for ≥25% of the time. PVI was assessed using multielectrode mapping catheters with a 1‐mm electrode size (Lasso Nav or PentaRay, Biosense Webster).

After completion of electrical PVI and restoration of sinus rhythm (with electrical cardioversion when needed), left atrial voltage mapping was performed during high right atrial pacing at 100 bpm. Mapping points were automatically acquired using the CONFIDENSE module (Biosense Webster) with multielectrode mapping catheters until all color gaps on the voltage map were filled, with the following settings: cycle length filtering, ±30 ms; local activation time stability, 3 ms; position stability, 2 mm; density, 1 mm; tissue proximity indicator, off; and fill and color interpolation threshold, 10 mm. Patients without protocol‐defined complete mapping were excluded before randomization. LVAs were defined as areas with bipolar peak‐to‐peak voltage <0.50 mV, and scar as <0.05 mV. LVA size was manually measured using the CARTO3 area measurement tool. Patients with LVA ≥5 cm^2^ were then randomized intraprocedurally to either PVI alone or PVI+LVA‐ABL.

In the PVI+LVA‐ABL group, adjunctive LVA ablation aimed to achieve homogenization of the LV substrate, excluding scar regions (<0.05 mV). Posterior LVAs could be isolated with roof and bottom lines. Each radiofrequency energy application was guided by the VISITAG Surpoint module with a target ablation index ≥350 and interlesion distance <6 mm. Ablation could be omitted at sites where it might impair the physiological conduction system or damage collateral structures, including the esophagus.

After protocol completion, the entrance and exit blocks were confirmed for the isolated pulmonary veins following a waiting period of at least 20 minutes. Atrial burst pacing was performed to induce atrial tachycardia (AT), including typical atrial flutter. Intravenous isoproterenol or adenosine triphosphate were administered to unmask pulmonary vein reconnection and to provoke nonpulmonary vein triggers, defined as reproducible premature atrial contractions that initiate AF. Ablation of reproducibly induced AT and nonpulmonary vein triggers was recommended, but all additional ablation beyond the assigned protocol was left to the operator's discretion.

### Follow‐Up and End Points

Patients were followed for 12 months with scheduled outpatient visits at 3, 6, and 12 months. At each visit, a standard 12‐lead ECG was obtained, and a 24‐hour Holter ECG (CM5 and NASA leads) was performed at 6 and 12 months. For 6 to 12 months, patients also performed twice‐daily handheld ECG recordings (30 seconds each) using a portable device (HCG 901 or HCG 801; Omron, Kyoto, Japan). Symptom‐triggered ECG recordings were encouraged to detect recurrent arrhythmias.

The primary efficacy end point was recurrence of AF or AT (AF/AT) within 12 months, excluding events during the 3‐month blanking period. AF/AT recurrences were defined as any atrial arrhythmia lasting ≥30 seconds identified ≥3 months after ablation; AF/AT episodes lasting <30 seconds on Holter ECG were considered clinically nonsignificant and were not defined as AF/AT recurrence. Antiarrhythmic drugs (AADs) were discouraged beyond 3 months in the absence of recurrence, and efficacy end points were assessed off antiarrhythmic therapy. The type of recurrence (AF versus AT) was adjudicated based on the first documented rhythm. AF was defined as an irregular atrial rhythm with fibrillatory waves on ECGs, whereas AT was defined as a regular atrial arrhythmia.

### Statistical Analysis

**Figure 1:**
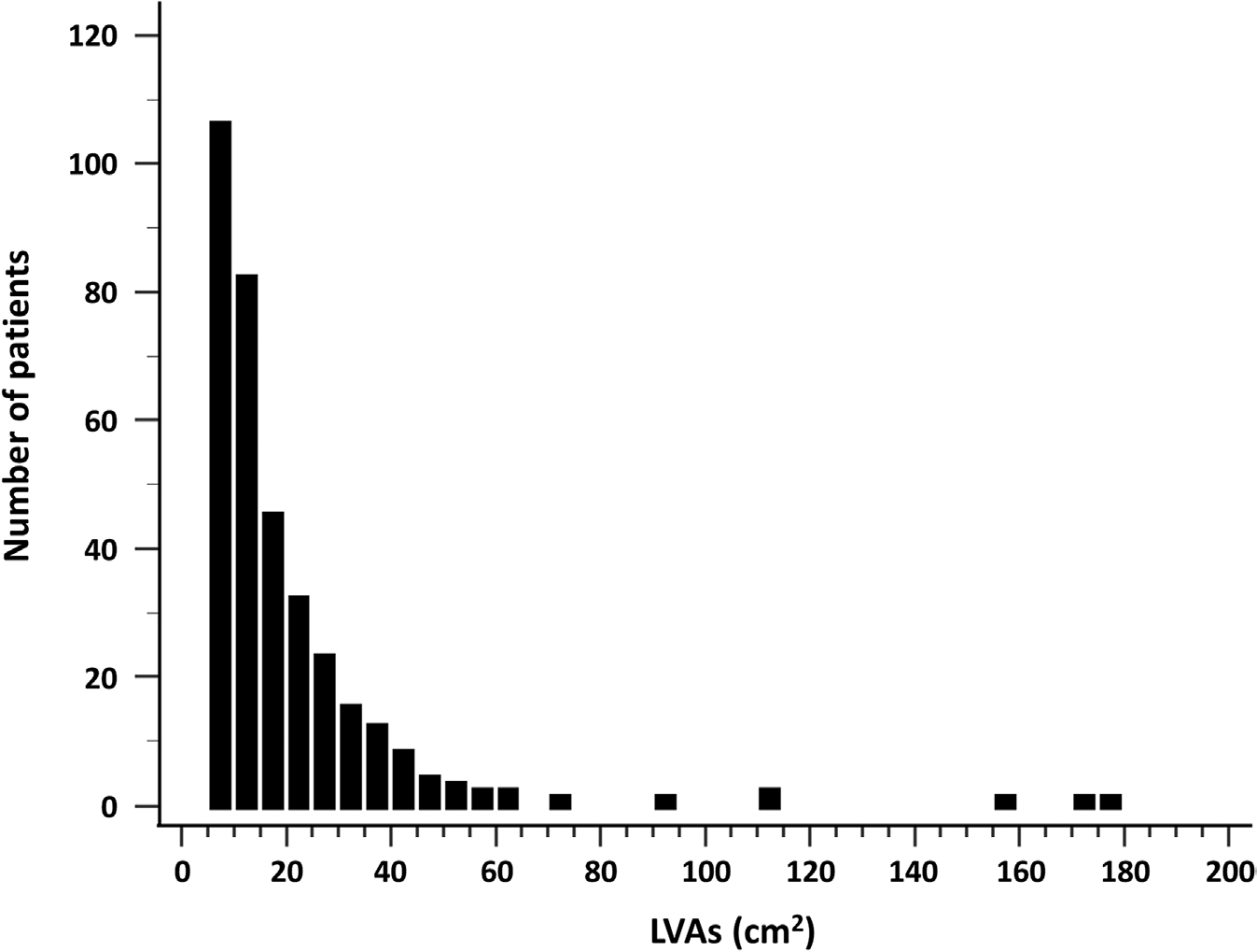
Distribution of LVA size. The distribution of left atrial LVA size was nonnormal (Shapiro–Wilk *P*<0.001) and right skewed, with a median of 13.3 cm^2^ (interquartile range, 8.7–23.2 cm^2^). LVA indicates low‐voltage area.

Continuous variables were expressed as mean ± SD or median (interquartile range), depending on their distribution. Categorical variables were presented as counts and percentages. The Shapiro–Wilk test was used to assess normality, particularly for LVA size, which showed a right‐skewed distribution (*P*<0.001; Figure 1). Accordingly, LVA size was analyzed using predefined size categories (small, moderate, extensive) or log‐transformed values, as appropriate. Trends across LVA groups were assessed using the Jonckheere–Terpstra test for continuous variables and the Cochran–Armitage trend test for categorical variables.

Freedom from AF/AT recurrence was estimated with the Kaplan–Meier method, and survival curves were compared using the log‐rank test. Treatment effects of PVI+LVA‐ABL versus PVI alone were evaluated using Cox proportional hazards models, and results were reported as hazard ratios (HRs) with 95% CIs. The interaction between treatment group and LVA size was also tested. Restricted cubic spline Cox models (knots at the 10th, 50th, and 90th percentiles) were used to explore continuous associations between LVA size and treatment effect.

Based on our prior left atrial size subanalysis,^15^ additional exploratory analyses were performed to contextualize LVA‐related findings with left atrial diameter. The association between left atrial diameter and LVA size was assessed using Pearson's correlation. For model comparisons, left atrial diameter was included as an adjustment term using restricted cubic splines, and alternative interaction specifications (treatment‐by‐LVA size, treatment‐by‐left atrial diameter, and both simultaneously) were compared using likelihood ratio tests and the Akaike information criterion. Heterogeneity of the treatment‐by‐LVA size relationship across left atrial diameter strata (>44 versus ≤44 mm) was evaluated using a three‐way interaction term (treatment × LVA size × left atrial diameter group).

Secondary analyses were performed to assess the type of recurrence (AF or AT), which was determined based on the first documented rhythm, as subsequent rhythms were not systematically collected. Event proportions by recurrence type were compared between treatment groups across LVA size categories, and exploratory treatment‐by‐LVA size interactions were evaluated using the same modeling approach. Complementary competing‐risk analyses were performed using Fine–Gray subdistribution hazard models.

All analyses were performed on a complete‐case basis without imputation of missing data. Statistical analyses were conducted with MedCalc, version 23 (MedCalc Software, Ostend, Belgium) and R version 4.4.2 (R Foundation for Statistical Computing, Vienna, Austria). A 2‐sided *P* < 0.05 was considered statistically significant.

## RESULTS

### Study Patients

Among the 341 study patients, the median LVA size was 13.3 cm^2^ (interquartile range, 8.7–23.2 cm^2^), with a right‐skewed distribution (*P*<0.001; Figure 1). Patients were stratified into 3 categories according to LVA size: small LVAs (≥5 to <10 cm^2^, n=106), moderate LVAs (≥10 to <20 cm^2^, n=127), and extensive LVAs (≥20 cm^2^, n=108).

Baseline characteristics across the LVA size categories are summarized in Table 1. Patients with larger LVAs were older, more often female, had higher stroke risk scores (CHADS_2_ and CHA_2_DS_2_‐VASc), and more frequently had heart failure, particularly heart failure with preserved ejection fraction. They also had lower hemoglobin levels, higher NT‐proBNP (N‐terminal prohormone of brain natriuretic peptide) levels, and lower estimated glomerular filtration rates. On echocardiography, left ventricular ejection fraction was similar across categories. Left atrial diameter increased modestly with LVA burden. However, the individual‐level association between left atrial diameter and LVA size was weak, consistent with our prior subgroup analysis (r=0.15, *P*=0.006; Figure S2).^15^ Diuretic use was more common in patients with extensive LVAs. Within each LVA size category, baseline characteristics were generally balanced between the PVI+LVA‐ABL and PVI‐alone groups, with only minor differences observed (Table S1).

**Table 1:** Baseline Characteristics

	Small LVA (≥5 to <10 cm^2^)	Moderate LVA (≥10 to <20 cm^2^)	Extensive LVA (≥20 cm^2^)	*P* for trend
	n=106	n=127	n=108	
Age, y	73 (69–77)	75 (70–79)	77 (72–80)	<0.001
Female sex, n (%)	41 (39)	60 (47)	66 (61)	0.001
Body mass index, kg/m^2^	23.7 (20.8–26.6)	23.6 (20.7–25.5)	22.7 (21.1–25.6)	0.377
Heart rate, beats per min	81 (69–91)	78 (70–90)	82 (71–93)	0.763
Systolic blood pressure, mm Hg	125 (114–136)	124 (111–135)	128 (118–141)	0.080
AF period, d	161 (76–486)	190 (90–945)	178 (93–628)	0.286
Duration of AF persistence, d	107 (59–304)	119 (66–313)	112 (62–294)	0.697
Long‐standing persistent AF*, n (%)	19 (18)	29 (23)	22 (20)	0.661
Comorbidities				
CHADS_2_ score	2 (1–2)	2 (1–3)	2 (1–3)	0.032
CHA_2_DS_2_‐VASc score	3 (2–4)	3 (3–4)	4 (3–5)	<0.001
Heart failure, n (%)	25 (24)	37 (29)	41 (38)	0.022
With preserved LVEF†	13 (12)	23 (18)	28 (26)	0.011
With reduced LVEF†	12 (11)	14 (11)	13 (12)	0.791
Hypertension, n (%)	71 (67)	93 (73)	78 (72)	0.401
Diabetes, n (%)	28 (26)	30 (24)	19 (18)	0.122
History of stroke or transient ischemic attack, n (%)	12 (11)	11 (8.7)	15 (14)	0.546
Laboratory data				
Hemoglobin, g/dL	13.9 (12.8–15.1)	13.6 (12.7–14.4)	13.3 (12.5–14.1)	0.001
N‐terminal prohormone of brain natriuretic peptide, pg/mL	955 (510–1593)	1090 (714–1586)	1219 (850–2000)	0.002
Estimated glomerular filtration rate, mL/min/1.73 m^2^	61.4 (52.8–72.7)	58.8 (48.4–68.4)	57.9 (47.9–68.4)	0.036
Echo data				
Left atrial diameter, mm	44.0 (38.9–47.1)	43.0 (39.2–48.0)	45.0 (42.5–48.0)	0.027
LVEF, %	59.3 (53.6–62.7)	57.6 (52.4–62.1)	58.0 (52.6–63.0)	0.907
Medications at discharge, n (%)				
Calcium blocker	44 (42)	65 (51)	47 (44)	0.775
Angiotensin‐converting enzyme inhibitor or angiotensin receptor blocker	38 (36)	56 (44)	44 (41)	0.470
Beta blocker	57 (54)	64 (50)	60 (56)	0.791
Diuretics	25 (24)	48 (38)	49 (45)	<0.001
Antiarrhythmic drugs, n (%)	34 (32)	50 (39)	30 (28)	0.499
Class I	9 (8.5)	17 (13)	4 (3.7)	0.211
Class III	25 (24)	33 (26)	26 (24)	0.936

*P* for trend is evaluated using Jonckheere's trend test for continuous variables and the Cochran–Armitage trend test for categorical variables.

AF indicates atrial fibrillation; LVA, low‐voltage area; and LVEF, left ventricular ejection fraction.

* Long‐standing persistent AF was defined as persistent AF lasting for >1 year.

^†^ LVEF was assessed by the Teichholz method; preserved EF was defined as LVEF ≥50% and reduced EF as LVEF <50%.

### Procedural Characteristics

Table 2 summarizes procedural characteristics by LVA size group. Although total procedure time was similar across the 3 groups, total ablation time and applied radiofrequency energy increased progressively with greater LVA burden (*P*=0.010 and *P*=0.001, respectively).

**Table 2:** Procedural Characteristics

	Small LVA (≥5 to <10 cm^2^)	Moderate LVA (≥10 to <20 cm^2^)	Extensive LVA (≥20 cm^2^)	*P* for trend
	n=106	n=127	n=108	
Total procedure time, min	170 (120–217)	170 (128–216)	175 (139–219)	0.232
Total ablation time, s	1735 (1371–2434)	1979 (1570–2453)	2022 (1602–2584)	0.010
Total applied radiofrequency energy, kJ	63.2 (52.7–81.3)	72.0 (55.9–86.9)	78.5 (59.0–99.9)	0.001
Deflectable sheath, n (%)	81 (76)	97 (76)	90 (83)	0.216
Mapping catheter				
Circular catheter, n (%)	12 (11)	20 (16)	12 (11)	0.958
Radiating catheter, n (%)	94 (89)	107 (84)	96 (89)	
Mapping points, n	1778 (1344–2525)	1643 (1251–2160)	1663 (1302–2288)	0.521
Mapping time, min	18.0 (12.6–25.0)	15.2 (13.0–21.0)	16.0 (12.0–20.9)	0.155
LVA size, cm^2^	7.1 (5.9–8.1)	13.3 (11.5–16.4)	29.8 (24.2–39.0)	<0.001
Left atrial surface area, cm^2^	155 (132–190)	164 (133–192)	167 (143–193)	0.097
LVA ablation	52 (49)	69 (54)	49 (45)	0.585
Applied energy, kJ	12.8 (8.9–20.1)	21.4 (14.4–28.4)	31.4 (22.3–38.3)	<0.001
Ablation time, s	385 (273–565)	582 (402–778)	768 (607–1061)	<0.001
Complete homogenization, n (%)	47 (90)	57 (83)	29 (59)	<0.001
Pulmonary vein isolation, n (%)	106 (100)	127 (100)	108 (100)	…
First‐pass isolation (left side), n (%)	94 (89)	113 (89)	93 (86)	0.562
First‐pass isolation (right side), n (%)	95 (90)	108 (85)	95 (88)	0.719
Nonpulmonary vein atrial fibrillation trigger ablation, n (%)	11 (10)	7 (5.8)	8 (7.4)	0.417
Superior vena cava, n (%)	5 (4.7)	0 (0.0)	3 (2.8)	0.359
Right atrium, n (%)	3 (2.8)	1 (0.8)	2 (1.9)	0.593
Left atrium, n (%)	3 (2.8)	6 (5.0)	3 (2.8)	0.979
Coronary sinus, n (%)	1 (0.9)	1 (0.8)	0 (0.0)	0.369
Cavotricuspid isthmus ablation	30 (28)	26 (21)	27 (25)	0.579
For clinical AFL, n (%)	5 (4.7)	4 (3.3)	1 (0.9)	0.103
For induced AFL, n (%)	24 (23)	22 (18)	25 (21)	0.923
As empirical ablation, n (%)	1 (0.9)	0 (0.8)	1 (0.9)	0.992
Ablation of regular AT, n (%)	6 (6.2)	14 (12)	20 (18)	0.008
Perimitral AT, n (%)	3 (2.8)	2 (1.7)	5 (4.7)	0.432
Roof‐dependent AT, n (%)	1 (0.9)	4 (3.1)	3 (2.8)	0.378
Biatrial AT, n (%)	0 (0.0)	1 (0.8)	1 (0.9)	0.376
Other ATs, n (%)	5 (4.7)	10 (7.9)	13 (12)	0.051
Periprocedural adverse events				
All events	2 (1.9)	5 (3.9)	8 (7.4)	0.049
Serious events	0 (0.0)	3 (2.5)	3 (2.8)	0.127
Cardiac tamponade, n (%)	0 (0.0)	1 (0.8)	0 (0.0)	1.000
Stroke or systemic embolism, n (%)	0 (0.0)	0 (0.0)	1 (0.9)	0.209
Esophageal fistula, n (%)	0 (0.0)	0 (0.0)	1 (0.9)	0.209
Major bleeding, n (%)	0 (0.0)	2 (1.7)	1 (0.9)	0.467
Death, n (%)	0 (0.0)	0 (0.0)	0 (0.0)	…

*P* for trend is evaluated using Jonckheere's trend test for continuous variables and the Cochran–Armitage trend test for categorical variables.

AFL indicates atrial flutter; AT, atrial tachycardia; and LVA, low‐voltage area.

Procedural characteristics differed between the PVI+LVA‐ABL and PVI‐alone groups within each LVA size category; however, these differences were attributable to the adjunctive LVA ablation itself rather than underlying electrophysiological variations (Table S2). In the PVI+LVA‐ABL group, complete LVA homogenization was less frequent in patients with extensive LVAs (29/49, 59%) than in those with small (47/52, 90%) or moderate (57/69, 83%) LVAs (*P*<0.001). The main reason for incomplete ablation was concern about injury to the esophagus or conduction systems including the His bundle and Bachmann's bundle (n=29). Other reasons included technical difficulty with catheter manipulation in the vicinity of the transseptal puncture site (n=4) and excessively extensive LVAs (n=3, Table S3).

There were no significant differences across LVA groups in the first‐pass isolation rate, the proportion of patients undergoing nonpulmonary vein trigger ablation or cavotricuspid isthmus ablation. However, ablation for regular AT was more frequently performed in patients with larger LVAs (*P* for trend =0.008). Periprocedural adverse events increased with LVA size (*P*=0.049), with the highest incidence observed in the group with extensive LVA (7.4%). Event rates did not differ significantly between PVI+LVA‐ABL and PVI‐alone strategies across LVA categories (Table S2). Pericarditis was observed in 0 (0%) patients in the PVI+LVA‐ABL group and 1 (0.6%) in the PVI‐alone group. However, an esophago‐pericardial fistula developed in one patient who underwent posterior wall isolation as part of adjunctive LVA ablation, despite multi‐sensor esophageal temperature monitoring with a 40 °C cutoff and prophylactic proton pump inhibitor therapy. The patient survived but experienced prolonged morbidity requiring pericardial drainage and multiple surgical interventions, with discharge only after an approximately 9‐month hospitalization.

### Efficacy End Points

**Figure 2:**
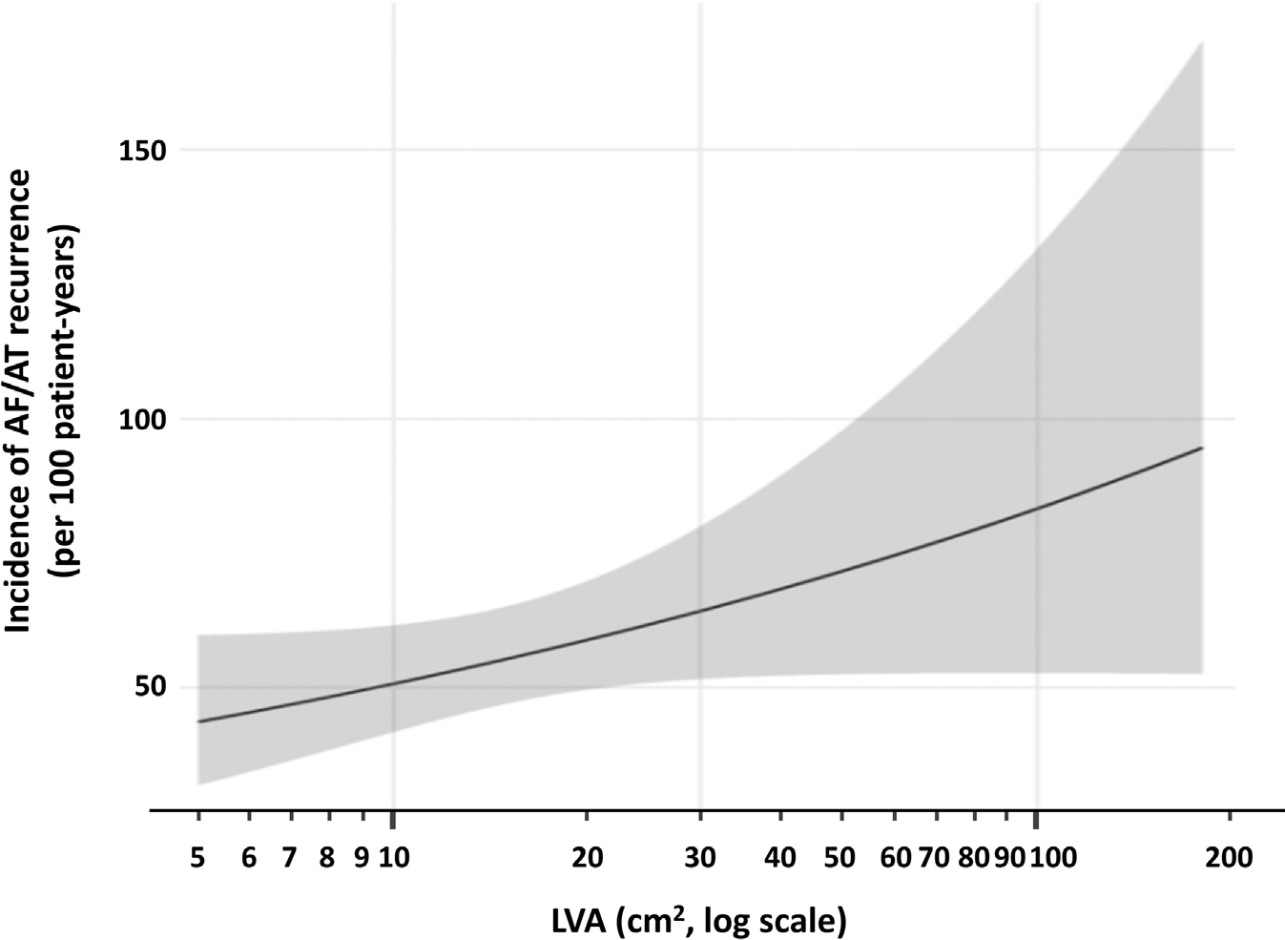
Incidence rate of AF/AT recurrence across LVA size. Association between LVA size and arrhythmia recurrence during follow‐up, evaluated using a Cox regression model with restricted cubic splines. The horizontal axis represents LVA size on a logarithmic scale, labeled with the corresponding absolute values (cm^2^); the vertical axis shows the adjusted incidence rate of AF/AT recurrence per 100 patient‐years. The incidence increased progressively with greater LVA burden. AF indicates atrial fibrillation; AT, atrial tachycardia; and LVA, low‐voltage area.

During the 12‐month follow‐up period, 17 patients dropped out because of missing daily ECGs or loss to outpatient follow‐up. The follow‐up protocol was completed in 324 (94.7%) patients. The primary end point, AF/AT recurrence in the absence of AADs, occurred in 148 of 341 patients (43.4%). Recurrence rates did not differ significantly across the 3 LVA size groups (small versus moderate versus extensive: 34.0% versus 46.5% versus 47.2%; log‐rank *P*=0.169; Figure S3). However, restricted cubic spline modeling demonstrated a positive association between LVA size and recurrence risk, with larger LVAs corresponding to higher AF/AT incidence per 100 patient‐years (*P* for overall <0.001, Figure 2).

Postablation AAD use during follow‐up is summarized in Table S4. AADs were prescribed to ∼10% of patients beyond the blanking period (3–12 months), with no significant difference between groups. Repeat ablation for recurrent arrhythmia was not performed within the 3‐month blanking period in either group; beyond the blanking period, it was performed in 23/170 (13.5%) patients in the PVI+LVA‐ABL group and 27/171 (15.8%) in the PVI‐alone group (*P*=0.556).

### Stratification by LVA Size Categories

**Figure 3:**
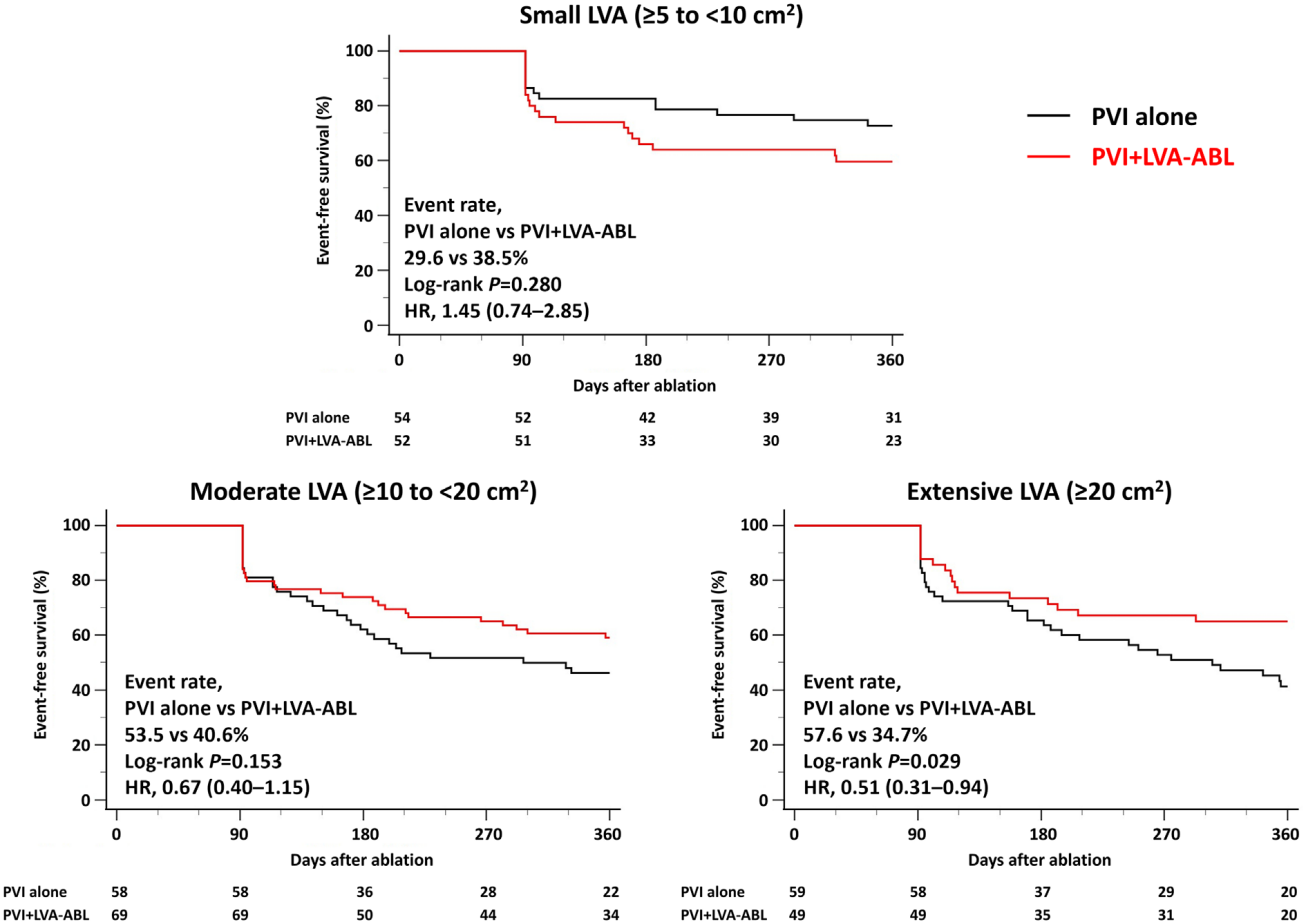
AF/AT‐recurrence‐free rate across LVA size categories. Kaplan–Meier curves showing freedom from AF/AT recurrence after a single procedure without antiarrhythmic drug use, stratified by LVA size category and treatment group. Adjunctive LVA ablation significantly reduced recurrence in patients with extensive LVAs but not in those with small or moderate substrate burden. AF indicates atrial fibrillation; AT, atrial tachycardia; HR, hazard ratio; LVA, low‐voltage area; PVI, pulmonary vein isolation; and PVI+LVA‐ABL, pulmonary vein isolation followed by low‐voltage area ablation.

Kaplan–Meier curves stratified by LVA size and treatment group are shown in Figure 3. In patients with small LVAs (≥5 to <10 cm^2^), AF/AT recurrence rates were similar between the PVI+LVA‐ABL and PVI‐alone (38.5% versus 29.6%; *P*=0.280; Figure 3A). Similarly, no significant difference was observed in the moderate LVA group (40.6% versus 53.5%; *P*=0.153; Figure 3B). However, in patients with extensive LVAs (≥20 cm^2^), recurrence was significantly lower in the PVI+LVA‐ABL group compared with PVI alone (34.7% versus 57.6%; *P*=0.029; Figure 3C). Although not statistically significant, there was a borderline interaction between treatment effect and LVA size category (*P* for interaction =0.054).

### Spline Analysis of Treatment Effect

**Figure 4:**
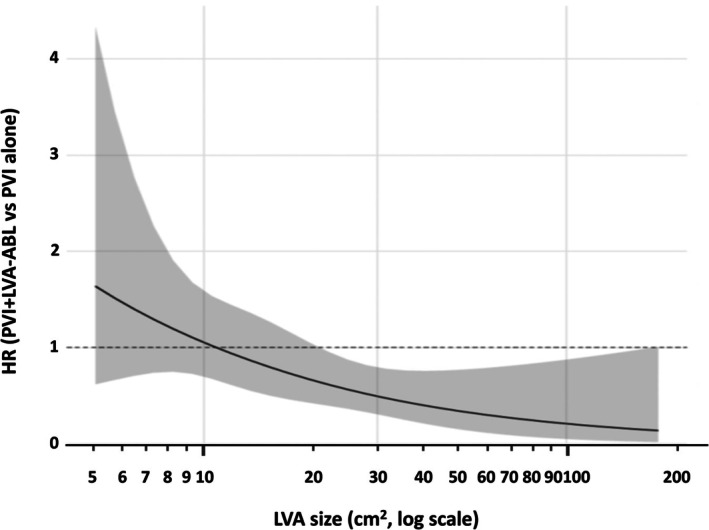
Treatment effect of LVA‐ABL compared with PVI alone across the spectrum of LVA size. Treatment effect of adjunctive LVA ablation across the continuous spectrum of LVA size, evaluated with a Cox regression model using restricted cubic splines. The horizontal axis represents LVA size on a logarithmic scale labeled with absolute values (cm^2^), and the vertical axis shows the estimated HR with 95% CIs (shaded area) for AF/AT recurrence. HR <1.0 indicates a favorable treatment effect of adjunctive LVA ablation compared with PVI alone. AF indicates atrial fibrillation; AT, atrial tachycardia; HR, hazard ratio; LVA, low‐voltage area; PVI, pulmonary vein isolation; and PVI+LVA‐ABL, pulmonary vein isolation followed by low‐voltage area ablation.

To evaluate the continuous relationship between LVA size and treatment effect, a Cox proportional hazards model with restricted cubic splines was constructed (Figure 4). The HR was >1.0 for smaller LVAs, crossed 1.0 at ∼10 cm^2^, and declined progressively with increasing LVA size. The upper bound of the 95% CI fell below 1.0 at approximately 20 cm^2^, indicating that the benefit became more consistently detectable beyond this range. For clinical interpretability, supplementary analyses were performed using binary LVA thresholds. When analyzed in 1‐cm^2^ increments, adjunctive LVA ablation demonstrated a consistent benefit from ≥7 cm^2^, with the strongest treatment‐by‐size interaction observed at 17 cm^2^ (*P* for interaction =0.008; Figure S4).

In exploratory model comparisons adjusted for left atrial diameter using restricted cubic splines, the treatment‐by‐LVA size interaction improved model fit (likelihood ratio test *P*=0.054), whereas the treatment‐by‐left atrial diameter interaction provided little additional improvement (*P*=0.249, Table S5). Nested comparisons further suggested a greater incremental contribution of the treatment‐by‐LVA size term than the treatment‐by‐left atrial diameter term (*P*=0.083 versus *P*=0.382; Table S5). The treatment‐by‐LVA size association did not differ by left atrial diameter strata (3‐way interaction *P*=0.556; Figure S5).

### Stratification by Initial Recurrence Pattern

**Figure 5:**
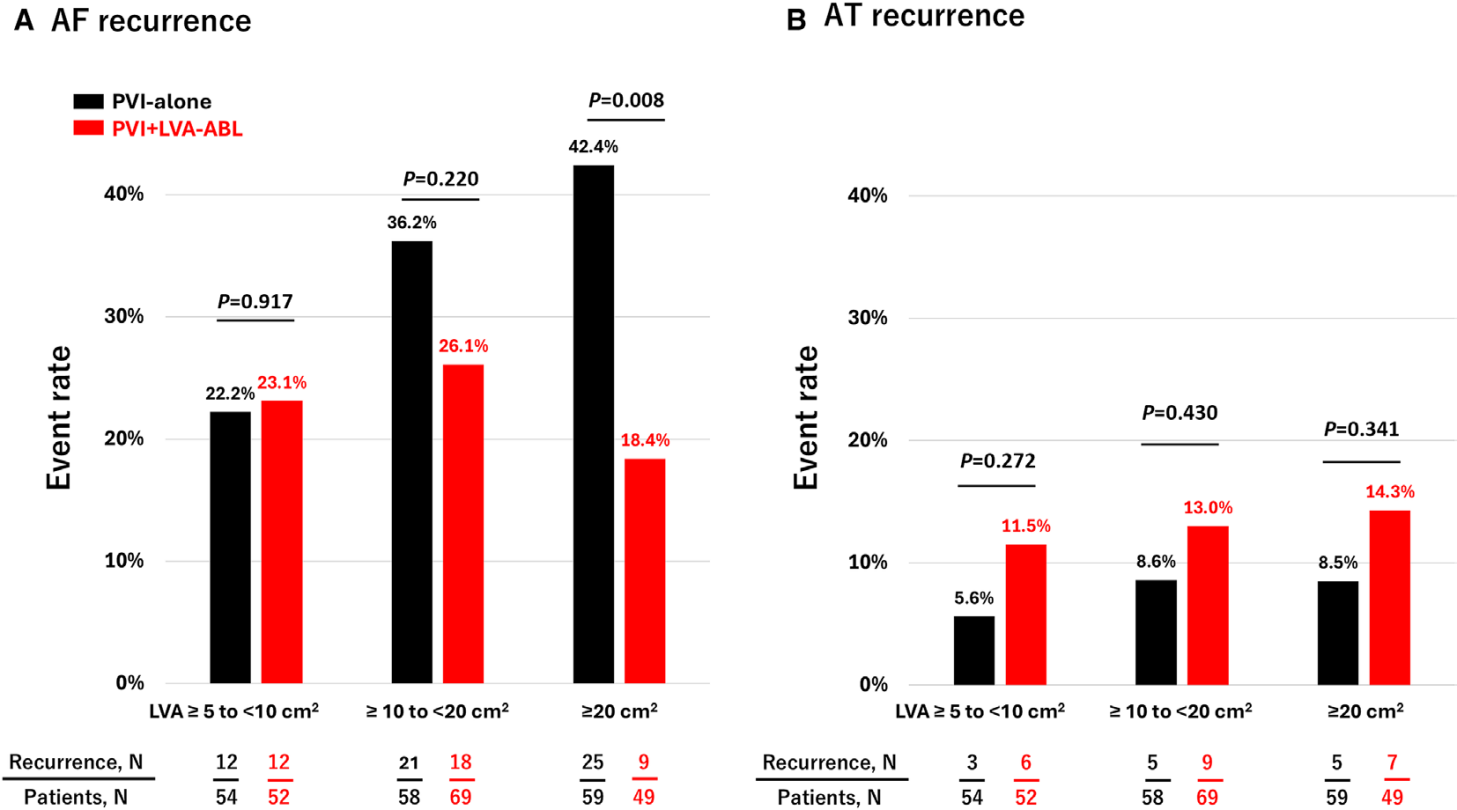
Type of arrhythmia recurrence across LVA size and treatment group. **A**, Proportions of patients with AF recurrence across treatment groups. **B**, Proportions of patients with AT recurrence across treatment groups. Because separate AF and AT recurrence dates were unavailable, recurrence type was defined by the first documented event after the blanking period. Event proportions during the 1‐y study period were compared between treatment groups within each LVA size category. AF indicates atrial fibrillation; AT, atrial tachycardia; LVA, low‐voltage area; PVI, pulmonary vein isolation; and PVI+LVA‐ABL, pulmonary vein isolation followed by low‐voltage area ablation.

**Figure 6:**
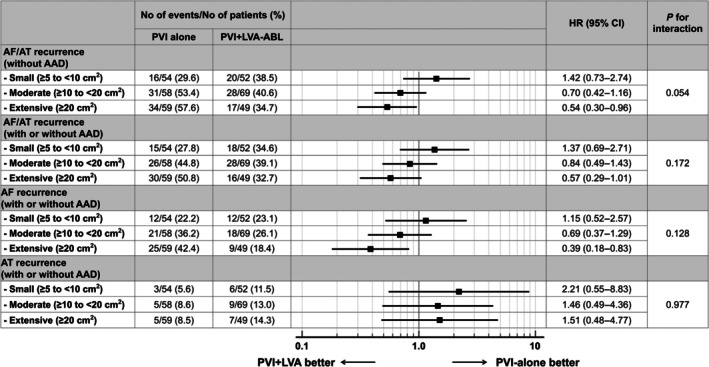
Treatment effect of PVI+LVA‐ABL compared with PVI alone across LVA size categories. Forest plot showing the treatment effect of adjunctive LVA ablation compared with PVI alone on 4 distinct end points: AF/AT recurrence without AADs, AF/AT recurrence, AF recurrence, and AT recurrence. Analyses were stratified by LVA size category (small, moderate, extensive), with HRs and 95% CIs displayed for each end point. AAD indicates antiarrhythmic drug; AF, atrial fibrillation; AT, atrial tachycardia; HR, hazard ratio; LVA, low‐voltage area; PVI, pulmonary vein isolation; and PVI+LVA‐ABL, pulmonary vein isolation followed by low‐voltage area ablation.

When recurrence was analyzed according to arrhythmia type, adjunctive LVA ablation significantly reduced AF recurrence in patients with extensive LVAs (*P*=0.008) but not in those with small or moderate LVAs (*P*=0.917 and *P*=0.220, respectively) (Figure 5A and Figure S6). In contrast, AT recurrence was slightly more frequent in patients with adjunctive LVA ablation, although the differences were not statistically significant across LVA size categories (Figure 5B and Figure S7). Although the interaction between treatment effect and LVA size was not statistically significant for either AF or AT recurrence, adjunctive LVA ablation appeared to reduce AF recurrence more effectively in patients with larger LVAs, whereas a slight increase in AT recurrence was observed across all LVA size categories (Figure 6).

## DISCUSSION

This post hoc analysis was conducted to complement the original SUPPRESS‐AF findings by examining the association between left atrial LVA size and the efficacy of adjunctive LVA ablation in patients with persistent AF. By combining categorical and spline‐based analyses, we quantitatively characterized the treatment effect across the entire LVA spectrum to better understand its mechanistic and clinical implications. The results demonstrated that the benefit of PVI+LVA ablation varied according to LVA size. Specifically, adjunctive LVA ablation significantly reduced arrhythmia recurrence in patients with extensive LVAs (≥20 cm^2^), whereas no clear benefit was observed in those with smaller LVAs. Spline analysis further indicated a progressive increase in treatment benefit with increasing LVA size. In interaction analyses, LVA size appeared to better capture the incremental benefit of substrate modification than left atrial diameter. These findings suggest that the efficacy of adjunctive LVA ablation is size dependent, which may partly explain the overall neutral results of the SUPPRESS‐AF trial and support the concept that patients with more advanced atrial remodeling, reflected by larger LVAs, are more likely to benefit from a PVI+LVA‐ABL strategy.

### Rationale for LVA Size‐Based Analysis in the SUPPRESS‐AF Trial

LVAs represent areas of advanced atrial tissue degeneration and are considered pivotal in the development and persistence of AF.^3^, ^4^, ^5^ Several randomized controlled trials have evaluated the efficacy of LVA ablation, but the SUPPRESS‐AF trial was unique in its design and patient selection. Unlike prior trials, SUPPRESS‐AF performed voltage mapping after PVI and enrolled only patients with confirmed LVAs (≥5 cm^2^), thereby ensuring that all randomized participants had a clearly defined arrhythmogenic substrate.

In contrast, earlier trials such as STABLE‐SR‐II^8^ (CPVI [Circumferential PVI] Alone Versus CPVI Plus Electrophysiological Substrate Ablation in the LA [Left Atrium] During SR [Sinus Rhythm] for the Treatment of Non‐PAF [Paroxysmal AF]) and ERASE‐AF^9^ (Low Voltage Guided Ablation Trial of Persistent Atrial Fibrillation) randomized patients before voltage mapping, leading to the inclusion of a substantial proportion of patients without LVAs. Indeed, LVAs were present in only 47.7% of patients in STABLE‐SR‐II (n=279) and 36.4% in ERASE‐AF trial (n=324). Consequently, the estimated median LVA size in those studies was close to 0 cm^2^, limiting their ability to assess treatment effects based on the substrate burden. By comparison, the median LVA size in SUPPRESS‐AF was 13.3 cm^2^ (interquartile range, 8.7–23.2 cm^2^), allowing for a more robust evaluation of size‐dependent treatment outcomes.

### Comparison of Previous Studies

The efficacy of LVA ablation across the spectrum of LVA size remains underexplored. Among prior randomized controlled trials, the STABLE‐SR‐II reported that patients with greater LVA burden (≥15% of left atrial surface) tended to benefit from adjunctive substrate ablation.^16^ Although only 18 patients exceeded this threshold, limiting the statistical power, the findings were generally consistent with those of the present study.

In contrast, the DECAAF II (Efficacy of Delayed Enhancement MRI [Magnetic Resonance Imaging]‐Guided Ablation vs Conventional Catheter Ablation of Atrial Fibrillation) trial, which stratified patients by fibrotic burden detected by magnetic resonance imaging, reported greater benefit of adjunctive ablation in patients with less extensive fibrosis.^17^ This discrepancy may reflect differences between magnetic resonance imaging‐detected fibrosis and LVAs identified by voltage mapping.^18^, ^19^ Magnetic resonance imaging ‐detected fibrosis represents dense collagen deposition, a largely irreversible marker of structural scarring, whereas LVAs identified by electroanatomical mapping denote heterogeneous, partially viable myocardium with slow conduction that may remain modifiable by ablation. Accordingly, patients with extensive LVAs but preserved conduction channels may still respond favorably to substrate homogenization, while those with diffuse nonconductive fibrosis may not.

The present SUPPRESS‐AF analysis, which employed the same voltage‐mapping approach as STABLE‐SR‐II, included a larger cohort of patients with substantial LVAs, thereby strengthening the evidence for a size‐dependent treatment effect. Restricted cubic spline analysis demonstrated that the HR for recurrence was >1.0 for small LVAs, crossed 1.0 at ∼10 cm^2^, and decreased thereafter. The upper bound of the 95% CI fell below 1.0 at ∼20 cm^2^, indicating that a statistically robust benefit of adjunctive LVA ablation became apparent beyond this threshold. Although the exact cutoff may vary depending on the study cohort, sample size, and LVA ablation design, the association between larger LVA size and greater reduction in arrhythmia recurrence was consistent in our study. Notably, if the enrollment criterion in the SUPPRESS‐AF trial had been set at ≥7 cm^2^ rather than ≥5 cm^2^, the overall trial results would have reached statistical significance (Figure S4). Such thresholds warrant validation through individual‐patient data meta‐analyses of randomized controlled trials.

### The Efficacy of LVA Ablation in Reducing AF Recurrence

Our analysis demonstrated that adjunctive LVA ablation significantly reduced AF recurrence in patients with extensive LVAs. Although complete substrate homogenization was often unachievable, primarily due to safety concerns, partial modification was still associated with meaningful reductions in recurrence. To optimize efficacy while minimizing unnecessary lesions, additional electrophysiological features, such as electrogram duration,^20^ signal fragmentation,^21^ anisotropic conduction,^22^ and decremental behavior,^23^ may help refine ablation targets, particularly in patients with very extensive LVAs.

From a pathological perspective, LVAs correspond to heterogeneous atrial remodeling, including fibrosis, increased intercellular space, myofibrillar loss, and decreased nuclear density.^4^ These alterations promote arrhythmogenesis through distinct mechanisms, such as slow conduction or electrogram fractionation. Although a direct causal relationship between these electrophysiological properties and AF persistence is difficult to establish, the arrhythmogenic potential appears inherent to LVAs. In patients with larger LVAs, adjunctive ablation may achieve greater debulking of arrhythmogenic substrate, which could explain the more pronounced reduction in AF recurrence observed in this subgroup.

### Mechanistic Interpretation of AT Recurrence

Although adjunctive LVA ablation effectively reduced AF recurrence, it was accompanied by a small increase in AT recurrence. Because systematic electroanatomic characterization at repeat procedures is not currently available, this trial cannot determine whether recurrent AT was predominantly substrate‐related or ablation‐related. Nonetheless, the recurrence patterns in Figure 5 provide context: AT recurrence increased with larger LVAs even in the PVI‐alone group, suggesting an important contribution of the underlying atrial substrate, while the absolute excess AT associated with adjunctive LVA ablation was modest (approximately 5%–6%) and did not show a clear size‐dependent gradient.

These findings support 2 nonmutually exclusive interpretations: (1) iatrogenic proarrhythmic mechanisms caused by newly created conduction barriers or gaps within ablated tissue; and (2) a competing‐risk phenomenon in which suppression of AF allows previously concealed AT circuits to become clinically manifest. In patients with smaller LVAs, iatrogenic substrate formation may have played a greater role, whereas in those with larger LVAs, the reduction in AF burden may have unmasked preexisting AT circuits. Thus, the apparent increase in AT recurrence likely reflects a complex interplay between procedural and pathophysiologic mechanisms rather than a purely adverse effect of LVA ablation. Future studies incorporating systematic remapping at repeat procedures may help clarify the predominant mechanism of recurrent AT.

### Implications for Lesion Design to Reduce AT Recurrence

An increased incidence of organized AT after substrate modification has also been reported in trials beyond the LVA‐guided strategy. The TAILORED‐AF (Tailored vs. Anatomical Ablation Strategy for Persistent Atrial Fibrillation) trial, which employed artificial intelligence–guided ablation targeting spatiotemporal dispersion, achieved a greater reduction in AF recurrence but was also associated with a higher incidence of organized AT requiring repeat ablation.^24^ Regarding LVA ablation, the VOLCANO (Catheter Ablation Targeting Low‐Voltage Areas After Pulmonary Vein Isolation in Paroxysmal Atrial Fibrillation Patients) trial reported increased AT after adjunctive LVA ablation in paroxysmal AF.^25^, ^26^ In contrast, the ERASE‐AF trial did not show an apparent increase in AT recurrence despite a more extensive ablation strategy.^9^ This discrepancy may relate, in part, to the lesion design used in ERASE‐AF, which anchored ablation lines to anatomic boundaries such as the mitral annulus or prior ablation lines, thereby minimizing residual conduction gaps and iatrogenic circuits.

Collectively, careful consideration of lesion design—particularly its continuity, completeness, and anchoring to anatomic boundaries—is essential to balance the antiarrhythmic efficacy of LVA ablation against the risk of creating new arrhythmogenic circuits.

### Safety Considerations

Procedural safety remains an important consideration, particularly in patients with extensive LVAs, who often have advanced atrial remodeling and require longer ablation and procedure times. Despite comparable rates of individual serious adverse events between treatment groups, overall complication rates were higher among patients with extensive LVAs. Notably, an esophago‐pericardial fistula occurred in one such patient undergoing posterior wall isolation despite multisensor esophageal temperature monitoring with termination at 40 °C. Although based on a single event and therefore imprecise, this corresponded to 1/170 (0.59%) in the PVI+LVA‐ABL group and 1/108 (0.93%) in patients with LVA ≥20 cm^2^, and is numerically higher than previous large registry estimates for radiofrequency ablation (0.038%).^27^ The patient survived but experienced prolonged morbidity requiring an ∼9‐month hospitalization.^11^ This highlights the need for meticulous energy delivery near vulnerable structures. Incorporating natural scar boundaries or preexisting low‐voltage regions into lesion design may reduce collateral injury without compromising procedural efficacy.^9^, ^28^

### Clinical Implications

This subanalysis demonstrated a clear benefit of adjunctive LVA ablation in patients with extensive LVAs. Although the cutoff suggested by spline analysis (∼20 cm^2^) remains exploratory, it may provide practical guidance for patient selection and procedural strategy in persistent AF. For patients below this threshold, routine LVA ablation may confer limited benefit and increase the risk of iatrogenic AT, suggesting that ablation limited to inducible AT may be sufficient. Conversely, in patients with extensive LVAs, LVA‐targeted substrate modification in addition to PVI may be justified even in the absence of inducible AT. Voltage mapping to quantify LVAs can therefore help tailor ablation strategies and refine patient selection in persistent AF.

### Limitations

Several limitations should be acknowledged. First, the SUPPRESS‐AF trial excluded patients with advanced left atrial enlargement (left atrial diameter ≥55 mm), prior cardiac surgery, and valvular AF; therefore, these findings may not be generalizable to patients with more advanced structural heart disease, who may have more extensive LVAs and a different efficacy–safety profile with substrate modification. Second, AF/AT recurrence was assessed using intermittent rhythm monitoring, raising the possibility that asymptomatic episodes were missed and that the absolute recurrence rates were underestimated. Longer continuous monitoring would have been informative but was not mandated to preserve feasibility and minimize patient burden in this pragmatic multicenter trial. With the same follow‐up protocol, missed episodes would be expected to occur similarly in both groups, potentially reducing the observed between‐group differences. In addition, recurrence type was classified by the first documented rhythm, and separate dates for AF and AT were unavailable, which may underestimate arrhythmia burden in analyses stratified by recurrence type. Third, although LVA size was measured using a standardized mapping protocol, interoperator variability or mapping density may have influenced quantification. In addition, quantitative metrics of PVI lesion‐set geometry and the use of adenosine triphosphate challenge were not systematically captured, precluding formal comparisons across groups. Fourth, the relatively low rate of complete homogenization in extensive LVAs may have underestimated the true potential of substrate modification in these patients. However, the main reasons for incomplete homogenization were concerns about collateral injury, including esophageal damage and impairment of atrial conduction (Table S3). Balancing procedural efficacy and safety is crucial in real‐world clinical practice. Finally, the efficacy of ablation in patients with very extensive LVAs remains uncertain. A prospective study including patients with scarring involving >60% of the atrium showed no additional benefit from homogenization, suggesting a potential plateau effect.^29^ Future large‐scale meta‐analyses are needed to address these unresolved questions.

## CONCLUSIONS

In this post hoc analysis of the SUPPRESS‐AF trial, the efficacy of adjunctive LVA ablation in persistent AF was closely associated with substrate size. Patients with extensive LVAs derived the greatest benefit from adjunctive ablation, whereas those with smaller LVAs had no additional advantage over PVI alone. These findings highlight the importance of individualized substrate assessment to optimize ablation strategies and improve outcomes in persistent AF.

## Sources of Funding

This work was supported by Biosense Webster Inc., through the investigator‐initiated study program IIS 510.

## Disclosures

Masuda M has received honoraria from Medtronic and Daiichi Sankyo. Minamiguchi H has received honoraria from Medtronic and Abbott, as well as technical guidance fees from Philips and Cook Medical. Inoue K has received honoraria from Johnson & Johnson. Sakata Y has received honoraria from Nippon Boehringer Ingelheim. All of these are unrelated to the present research. The remaining authors have no disclosures to report.

## Supporting information

Data S1. The Osaka Cardiovascular Conference (OCVC)‐Arrhythmia Investigators
Tables S1–S5
Figures S1–S7

CONSORT Checklist
