# Supplementary material for: Efficacy of Low‐Voltage Area Ablation Across Substrate Size in Persistent Atrial Fibrillation: A Post Hoc Analysis of the SUPPRESS‐AF Randomized Trial
Source: J Am Heart Assoc. 2026 May 25;15(11):e047814. doi: 10.1161/JAHA.125.047814 (PMC13315134; doi:10.1161/JAHA.125.047814)
Supplement: Supplementary file 1 — Data S1. The Osaka Cardiovascular Conference (OCVC)‐Arrhythmia Investigators Tables S1–S5 Figures S1–S7 [file JAH3-15-e047814-s001.pdf]

SUPPLEMENTAL MATERIAL

Data S1. The Osaka Cardiovascular Conference (OCVC)-Arrhythmia Investigators

Chair: Yasushi Sakata, Department of Cardiovascular Medicine, Osaka University Graduate School of Medicine, 2-2 Yamada-oka, Suita 565-0871, Japan

Secretariat: Yohei Sotomi (Chief), Daisaku Nakatani, Katsuki Okada, Tomoharu Dohi, Akihiro Sunaga, Hirota Kida, Yuki Matsuoka, and Daisuke Sakamoto; Department of Cardiovascular Medicine, Osaka University Graduate School of Medicine, Suita, Japan.

Investigators: Masato Okada, Nobuaki Tanaka, Koji Tanaka, Yuko Hirao, and Katsuomi Iwakura, Sakurabashi Watanabe Advanced Healthcare Hospital, Osaka, Japan; Masaharu Masuda, Yasuhiro Matsuda, Hiroyuki Uematsu, and Toshiaki Mano, Kansai Rosai Hospital, Amagasaki, Japan; Nobuhiko Makino, Hitoshi Minamiguchi, Takashi Kanda, Akio Hirata, and Yoshiharu Higuchi, Osaka Keisatsu Hospital, Osaka, Japan; Yasuyuki Egami, Masamichi Yano, Yasuharu Matsunaga-Lee, and Masami Nishino, Osaka Rosai Hospital, Sakai, Japan; Masato Kawasaki, Mitsutoshi Asai, and Takahisa Yamada, Osaka General Medical Center, Osaka, Japan; Tetsuya Watanabe, Tomoko Minamisaka and Shiro Hoshida, Yao Municipal Hospital, Yao, Japan; Koichi Inoue, Tsuyoshi Mishima, Tatsuhisa Ozaki, Takuya Ohashi, and Yasunori Ueda, National Hospital Organization Osaka National Hospital, Osaka, Japan; and Yasushi Sakata, Shungo Hikoso, Daisaku Nakatani, Hiroya Mizuno, Toshihiro Takeda, Takafumi Oka, Tomoaki Nakano, Kentaro Ozu, Takayuki Sekihara, Katsuki Okada, Tomoharu Dohi, Yohei Sotomi, Akihiro Sunaga, Hirota Kida, Bolrathanak Oeun, Taiki Sato, Yuki Matsuoka, and Daisuke Sakamoto, Osaka University Graduate School of Medicine, Suita, Japan

Table S1. Baseline characteristics

	Small LVA (≥5 to <10 cm^2)			Moderate LVA (≥10 to <20 cm^2)			Extensive LVA (≥20 cm^2)		
	PVI-alone n = 54	PVI+LVA-ABL n = 52	P value	PVI-alone n = 58	PVI+LVA-ABL n = 69	P value	PVI-alone n = 59	PVI+LVA-ABL n = 49	P value
Age, years	73 (50, 84)	73 (57, 87)	0.962	76 (72, 79)	74 (69, 79)	0.228	77 (73, 80)	75 (71, 79)	0.129
Female, n (%)	19 (35)	22 (42)	0.454	30 (52)	30 (44)	0.356	33 (56)	33 (67)	0.228
Body mass index, kg/m^2	23.7 (20.8, 26.6)	23.8 (20.7, 25.5)	0.975	23.5 (20.7, 25.0)	23.6 (20.8, 26.2)	0.934	23.7 (20.8, 26.6)	23.6 (20.7, 25.5)	0.377
Heart rate, beats per min	83 (69, 96)	79 (70, 88)	0.190	78 (71, 90)	79 (69, 90)	0.862	81 (69, 91)	78 (70, 90)	0.763
Systolic blood pressure, mmHg	125 (111, 136)	125 (115, 135)	0.700	125 (114, 138)	120 (110, 134)	0.272	125 (114, 136)	124 (111, 135)	0.080
AF period, days	157 (76, 593)	226 (74, 462)	0.850	182 (87, 658)	233 (99, 968)	0.304	229 (102, 760)	150 (72, 611)	0.275
Duration of AF persistence, days	115 (66, 234)	94 (56, 347)	0.832	102 (53, 254)	130 (75, 363)	0.175	115 (59, 295)	107 (62, 280)	0.941
Long-standing persistent AF, n (%)	7 (13)	12 (23)	0.177	13 (22)	16 (23)	0.918	12 (20)	10 (20)	0.993
**Comorbidities**									
$CHADS_2$ score	2 (1, 2)	2 (1, 2)	0.870	2 (1, 2)	2 (1, 3)	0.392	2 (1, 3)	2 (1, 3)	0.954
CHA_2DS_2-VASc score	3 (2, 4)	3 (2, 4)	0.849	4 (3, 5)	3 (2, 4)	0.074	4 (3, 4)	4 (3, 4)	0.795
Heart failure, n (%)	15 (28)	10 (19)	0.302	15 (26)	22 (32)	0.459	24 (41)	17 (35)	0.525
- with preserved LVEF*	9 (17)	4 (7.7)	0.161	11 (19)	12 (17)	0.819	19 (32)	9 (18)	0.104
- with reduced LVEF*	6 (11)	6 (12)	0.945	4 (6.9)	10 (14)	0.175	5 (8.5)	8 (16)	0.214
Hypertension, n (%)	36 (67)	35 (67)	0.944	42 (72)	51 (74)	0.850	39 (66)	39 (80)	0.121
Diabetes mellitus, n (%)	14 (26)	14 (27)	0.908	12 (21)	18 (26)	0.477	9 (15)	10 (20)	0.486
History of Stroke or TIA, n (%)	5 (9.3)	7 (14)	0.497	8 (14)	3 (4.3)	0.110	9 (15)	6 (12)	0.654
**Laboratory data**									
Hemoglobin, g/dL	13.9 (13.0, 15.0)	13.8 (12.7, 15.3)	0.827	13.5 (12.5, 14.3)	13.7 (12.7, 14.7)	0.401	13.3 (12.4, 14.1)	13.3 (12.5, 14.3)	0.925
NT-pro BNP, pg/mL	902 (499, 1215)	1188 (741, 1582)	0.166	1062 (696, 1591)	1108 (715, 1618)	0.858	1198 (821, 1927)	1469 (859, 2318)	0.305
eGFR, mL/min/1.73m^2	60.5 (52.8, 68.7)	63.8 (52.8, 78.9)	0.229	57.6 (52.1, 67.7)	60.1 (47.1, 71.5)	0.709	58.4 (48.5, 71.0)	56.1 (45.5, 65.5)	0.259

**Echo data**									
Left atrial diameter, mm	42.5 (39.0, 47.0)	45.0 (38.5, 47.7)	0.574	44.0 (38.9, 47.1)	43.0 (39.2, 48.0)	0.027	45.0 (42.5, 48.0)	45.0 (42.6, 48.0)	0.665
LVEF, %	59.3 (55.6, 62.9)	60.2 (51.3, 62.7)	0.767	57.8 (52.4, 63.0)	56.6 (52.3, 61.9)	0.694	58.7 (53.9, 64.5)	57.2 (51.4, 61.9)	0.176
**Medications at discharge, n (%)**									
Calcium blocker	21 (39)	23 (44)	0.579	29 (50)	36 (52)	0.808	25 (42)	22 (45)	0.793
ACE-I or ARB	17 (32)	18 (35)	0.733	23 (40)	33 (48)	0.358	22 (37)	22 (45)	0.425
Beta blocker	26 (48)	31 (60)	0.239	26 (45)	38 (55)	0.252	36 (61)	24 (49)	0.212
Diuretics	10 (19)	15 (29)	0.213	17 (29)	31 (45)	0.072	24 (41)	25 (51)	0.285
Antiarrhythmic drugs, n (%)	19 (35)	15 (29)	0.487	23 (40)	27 (39)	0.952	17 (29)	13 (27)	0.793
- Class I	8 (15)	1 (1.9)	0.032	8 (14)	9 (13)	0.902	2 (3.4)	2 (4.1)	1.000
- Class III	11 (20)	14 (27)	0.429	15 (26)	18 (26)	0.977	15 (25)	11 (22)	0.720

Continuous variables were expressed as mean ± standard deviation or median (interquartile range), depending on their distribution. Categorical variables were presented as counts and percentages. Comparisons between the PVI-alone and PVI+LVA-ABL groups were performed using the Mann–Whitney U test for continuous variables and the chi-square or Fisher exact test for categorical variables, as appropriate.

* LVEF was assessed by the Teichholz method; preserved ejection fraction was defined as LVEF ≥50% and reduced ejection fraction as LVEF <50%.

ACE-I, angiotensin-converting enzyme inhibitor; AF, atrial fibrillation; ARB, angiotensin receptor blockers; NYHA, New York Heart Association; NT-pro BNP, N-terminal prohormone of brain natriuretic peptide; eGFR, estimated glomerular filtration rate; LVA, low-voltage area; LVEF, left ventricular ejection fraction; TIA, transient ischemic attack

Table S2. Procedural characteristics

	Small LVA (≥5 to <10 cm²)			Moderate LVA (≥10 to <20 cm²)			Extensive LVA (≥20 cm²)		
	**PVI-alone n = 54**	**PVI+LVA-ABL n = 52**	**P value**	**PVI-alone n = 58**	**PVI+LVA-ABL n = 69**	**P value**	**PVI-alone n = 59**	**PVI+LVA-ABL n = 49**	**P value**
**Total procedure time, min**	150 (118, 185)	173 (129, 240)	0.017	164 (116, 189)	180 (136, 226)	0.020	166 (128, 213)	183 (141, 231)	0.217
**Total ablation time, sec**	1544 (1279, 2109)	2127 (1537, 2597)	<0.001	1605 (1338, 1983)	2218 (1901, 3078)	0.010	1742 (1387, 2194)	2517 (2121, 3022)	<0.001
**Total applied radiofrequency energy, kJ**	56.5 (46.3, 70.0)	71.5 (56.9, 90.3)	0.001	57.4 (48.8, 71.9)	80.6 (70.6, 105)	0.001	62.4 (51.6, 79.1)	91.8 (78.4, 110)	<0.001
**Deflectable sheath, n (%)**	43 (80)	38 (73)	0.429	44 (76)	53 (77)	0.901	47 (80)	43 (88)	0.263
**Mapping catheter**									
Circular catheter, n (%)	6 (11)	6 (12)	0.945	9 (16)	11 (16)	0.948	6 (10)	6 (12)	0.734
Radiating catheter, n (%)	48 (89)	46 (88)		49 (84)	58 (84)		53 (90)	43 (88)	
**Mapping points, n**	1800 (1394, 2463)	1671 (1261, 2525)	0.647	1512 (1188, 2049)	1708 (1308, 2187)	0.521	1667 (1278, 2291)	1651 (1318, 2278)	0.786
**Mapping time, min**	15.9 (12.0, 23.0)	19.0 (14.0, 25.0)	0.123	15.0 (13.0, 20.0)	16.0 (12.6, 21.5)	0.414	15.9 (12.0, 21.0)	16.0 (10.3, 20.0)	0.427
**LVA size, cm²**	7.1 (6.0, 8.2)	7.1 (5.9, 8.1)	0.714	13.3 (11.1, 16.4)	13.3 (11.6, 16.4)	0.588	27.5 (23.2, 37.2)	31.6 (25.4, 41.4)	0.251
**Left atrial surface area size, cm²**	154 (129, 177)	157 (135, 195)	0.266	164 (133, 192)	155 (132, 190)	0.097	160 (141, 190)	169 (148, 195)	0.360
**LVA ablation**	-	52 (100)	-	-	69 (100)	-	-	49 (100)	-
Applied energy, kJ	-	12.8 (8.9, 20.1)	-	-	21.4 (14.4, 28.4)	-	-	31.4 (22.3, 38.3)	-
Ablation time, sec	-	385 (273, 565)	-	-	582 (402, 778)	-	-	768 (607, 1061)	-
Complete homogenization, n (%)	-	47 (90)	-	-	57 (83)	-	-	29 (59)	-
**Pulmonary vein isolation, n (%)**	54 (100)	52 (100)	-	58 (100)	69 (100)	-	59 (100)	49 (100)	-
First pass isolation (left side), n (%)	44 (82)	50 (96)	0.029	53 (91)	60 (87)	0.430	52 (88)	41 (84)	0.506
First pass isolation (right side), n (%)	47 (87)	48 (92)	0.376	51 (88)	57 (83)	0.404	53 (90)	42 (86)	0.515
**Non-PV AF trigger ablation, n (%)**	7 (13)	4 (7.7)	0.527	4 (6.9)	3 (4.3)	0.701	6 (10)	2 (4.1)	0.288
Superior vena cava, n (%)	3 (5.6)	2 (3.8)	1.000	0 (0.0)	0 (0.0)	1.000	2 (3.4)	1 (2.0)	1.000

Right atrium, n (%)	2 (3.7)	1 (1.9)	1.000	0 (0.0)	1 (1.4)	1.000	2 (3.4)	0 (0.0)	0.500
Left atrium, n (%)	1 (1.9)	2 (3.8)	0.614	4 (6.9)	2 (2.8)	0.411	2 (3.4)	1 (2.0)	1.000
Coronary sinus, n (%)	1 (1.9)	0 (0.0)	1.000	0 (0.0)	1 (1.4)	1.000	0 (0.0)	0 (0.0)	1.000
**Cavo-tricuspid isthmus ablation**	13 (24)	17 (33)	0.327	15 (26)	11 (16)	0.169	12 (20)	15 (31)	0.222
for clinical AFL, n (%)	2 (3.7)	3 (5.8)	0.675	1 (1.7)	3 (4.3)	0.625	1 (1.7)	0 (0.0)	1.000
for induced AFL, n (%)	10 (19)	14 (27)	0.304	14 (24)	8 (12)	0.064	11 (19)	14 (29)	0.923
as empirical ablation, n (%)	1 (1.9)	0 (0.0)	1.000	0 (0.0)	0 (0.0)	0.992	0 (0.0)	1 (2.0)	0.273
**Ablation of regular AT, n (%)**	1 (1.9)	5 (9.6)	0.109	5 (8.6)	9 (13)	0.430	6 (10)	14 (29)	0.015
Perimitral AT, n (%)	1 (1.9)	2 (3.8)	0.614	2 (3.4)	3 (4.3)	0.432	2 (3.4)	3 (6.1)	0.432
Roof-dependent AT, n (%)	1 (1.9)	0 (0.0)	1.000	0 (0.0)	4 (5.8)	0.064	2 (3.4)	1 (2.0)	1.000
Biatrial AT, n (%)	0 (0.0)	0 (0.0)	1.000	0 (0.0)	1 (1.4)	1.000	0 (0.0)	1 (2.0)	0.273
Other ATs, n (%)	1 (1.9)	4 (7.7)	0.201	5 (8.6)	5 (7.2)	0.775	4 (6.8)	9 (18)	0.067
**Periprocedural adverse events**									
**All events**	0 (0.0)	2 (3.8)	0.238	1 (1.7)	4 (5.8)	0.375	3 (5.1)	5 (10)	0.314
**Serious events**	0 (0.0)	0 (0.0)	-	1 (1.7)	2 (2.9)	1.00	2 (3.4)	1 (2.0)	0.127
Cardiac tamponade, n (%)	0 (0.0)	0 (0.0)	-	1 (1.7)	0 (0.0)	1.00	0 (0.0)	0 (0.0)	-
Stroke or systemic embolism, n (%)	0 (0.0)	0 (0.0)	-	0 (0.0)	0 (0.0)	-	1 (1.7)	0 (0.0)	0.209
Esophageal fistula, n (%)	0 (0.0)	0 (0.0)	-	0 (0.0)	0 (0.0)	-	0 (0.0)	1 (2.0)	0.273
Major bleeding, n (%)	0 (0.0)	0 (0.0)	-	0 (0.0)	2 (2.9)	0.500	1 (1.7)	0 (0.0)	0.467
Death, n (%)	0 (0.0)	0 (0.0)	-	0 (0.0)	0 (0.0)	-	0 (0.0)	0 (0.0)	-

Continuous variables were expressed as mean ± standard deviation or median (interquartile range), depending on their distribution. Categorical variables were presented as counts and percentages. Comparisons between the PVI-alone and PVI+LVA-ABL groups were performed using the Mann–Whitney U test for continuous variables and the chi-square or Fisher exact test for categorical variables, as appropriate.

AFL, atrial flutter; AT, atrial tachycardia; LVA, low-voltage area; PVI, pulmonary vein isolation

Table S3. Reasons for incomplete LVA homogenization

	Small LVA (≥5 to <10 cm^2) n = 52	Moderate LVA (≥10 to <20 cm^2) n = 69	Extensive LVA (≥20 cm^2) n = 49	P for trend
**LVA ablation**				
Complete homogenization, n (%)	47 (90)	57 (83)	29 (59)	<0.001
Incomplete homogenization, n (%)	5 (9.6)	12 (17)	20 (41)	
**Reasons for incomplete homogenization**				
Concerns about collateral injury or atrial conduction	4 (7.7)	11 (16)	14 (29)	0.005
Technical difficulty with catheter manipulation	1 (1.9)	1 (1.4)	2 (4.0)	0.482
Extensive LVAs	0 (0.0)	0 (0.0)	3 (6.1)	0.021
Prolonged procedure time	0 (0.0)	0 (0.0)	1 (2.0)	0.185

Data were expressed as counts and percentages. P for trend is evaluated using the Cochran–Armitage trend test.

LVA, low-voltage area

Table S4. Post-ablation antiarrhythmic drug use

	PVI+LVA-ABL	PVI-alone	P value
**At discharge**	**n = 170**	**n = 171**	
All, n (%)	55 (32.4)	59 (34.5)	0.674
Class I, n (%)	12 (7.1)	18 (10.5)	0.259
Class III, n (%)	43 (25.3)	41 (24.0)	0.778
**At 3 months**	**n = 162**	**n = 163**	
All, n (%)	27 (16.7)	21 (12.9)	0.337
Class I, n (%)	4 (2.5)	4 (2.5)	0.993
Class III, n (%)	23 (14.2)	17 (10.4)	0.302
**At 6 months**	**n = 166**	**n = 165**	
All, n (%)	21 (12.7)	18 (10.9)	0.624
Class I, n (%)	1 (0.6)	3 (1.8)	0.371
Class III, n (%)	20 (12.0)	15 (9.1)	0.382
**At 12 months**	**n = 158**	**n = 159**	
All, n (%)	20 (12.7)	17 (10.7)	0.586
Class I, n (%)	3 (1.9)	4 (2.5)	1.000
Class III, n (%)	17 (10.8)	13 (8.2)	0.433

Medication use was assessed at scheduled follow-up visits. Denominators represent patients with available medication data at each time point; therefore, denominators vary due to missing visit/medication records.

LVA, low-voltage area; PVI, pulmonary vein isolation

Table S5. Likelihood-based model comparisons for effect modification by LVA size and left atrial diameter

(A) Comparison with the base model (no interaction terms)

Model	Interaction term(s) added	df	ΔLR χ^2	P (LR test)	AIC
**Base**	none	–	–	–	1602.45
**Model 1**	Treatment × LVA size	2	5.826	0.054	1600.63
**Model 2**	Treatment × LAD	2	2.783	0.249	1603.67
**Model 3**	Treatment × LVA size + Treatment × LAD	4	7.754	0.101	1602.70

(B) Incremental contribution of each interaction term (nested comparisons)

Nested comparison	Added term	df	ΔLR χ^2	P (LR test)
**Model 1 → Model 3**	+ (Treatment × LAD)	2	1.928	0.382
**Model 2 → Model 3**	+ (Treatment × LVA size)	2	4.970	0.083

*In Panel A, ΔLR χ^2 and P values are from likelihood ratio (LR) tests comparing each model with the base model. In Panel B, ΔLR χ^2 and P values quantify the incremental improvement in fit when adding the specified interaction term to the nested model.

Treatment indicates randomized treatment strategy (PVI plus LVA ablation vs. PVI alone). LVA size was modeled on a log scale using restricted cubic splines (3 knots). LAD was modeled using restricted cubic splines (3 knots). A lower Akaike information criterion indicates better model fit.

AIC, Akaike information criterion; LAD, left atrial diameter; LR, likelihood ratio; LVA, low-voltage area; PVI, pulmonary vein isolation.

Figure S1. Study flow

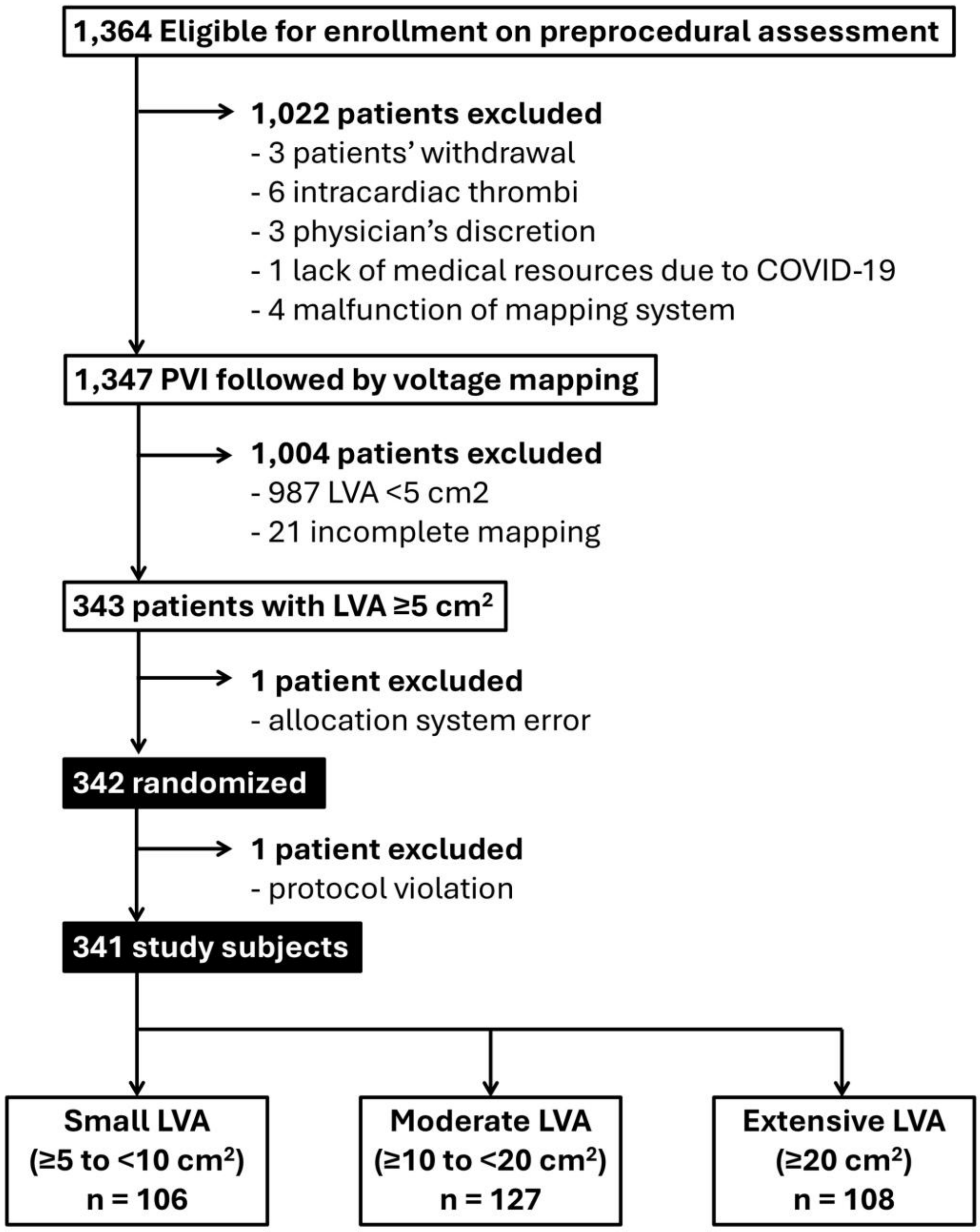

LVA, low-voltage area; PVI, pulmonary vein isolation

Figure S2. Association between left atrial diameter and LVA size

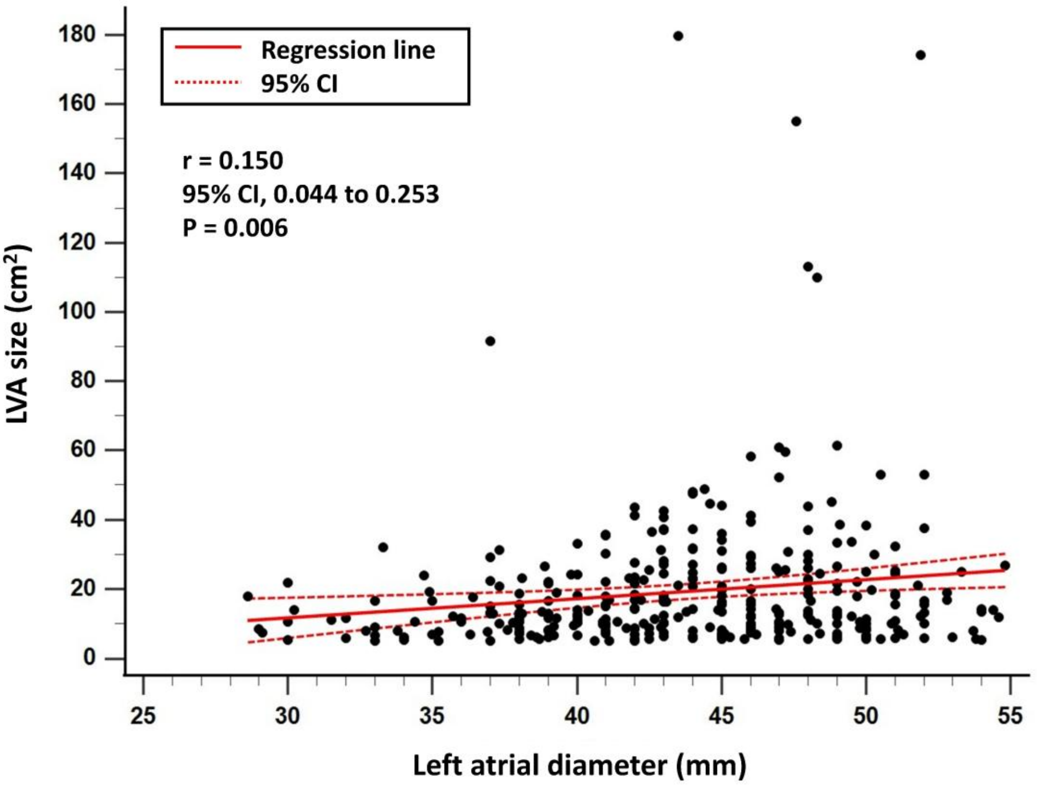

CI, confidence interval; LVA, low-voltage area.

Figure S3. Kaplan-Meier curves for freedom from AF/AT recurrence without antiarrhythmic drugs across LVA size categories

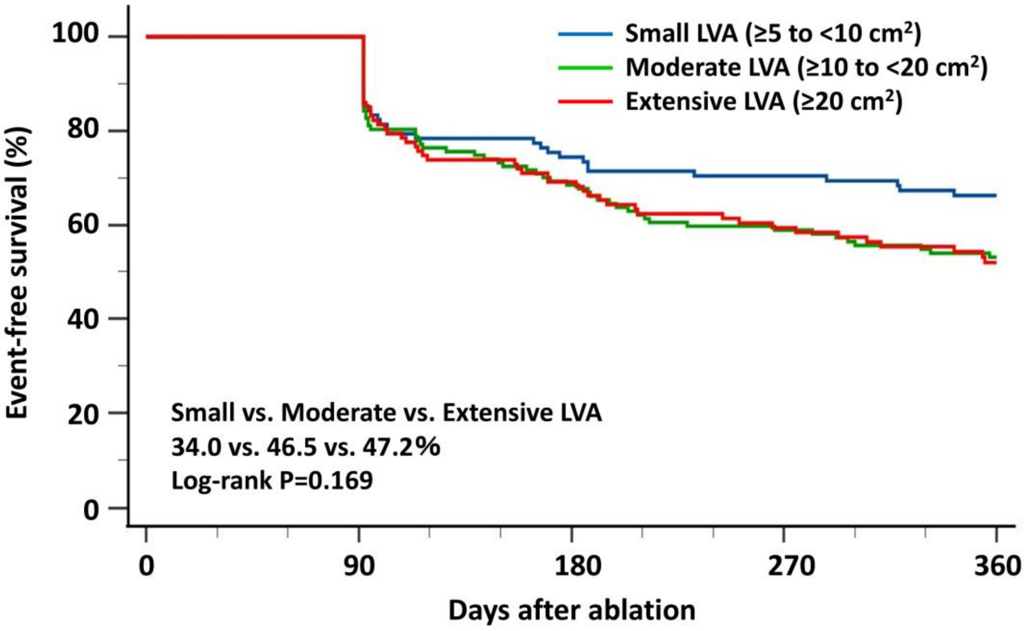

Small LVA	106	103	75	69	54
Moderate LVA	127	127	86	72	56
Extensive LVA	108	107	72	60	40

LVA, low-voltage area; AF, atrial fibrillation; AT, atrial tachycardia

Figure S4. Forest plots of treatment effect according to LVA size

LVA size subgroups	No of events/No of patients (%)		HR (95% CI)	P for interaction
	PVI alone	PVI+LVA ablation		
≥6 cm2	78/158 (49.4)	61/156 (39.1)	0.76 (0.54-1.06)	0.426
<6 cm2	3/13 (23.1)	4/14 (28.6)	1.31 (0.29-5.86)	
≥7 cm2	74/146 (50.7)	55/145 (37.9)	0.70 (0.49-0.99)	0.101
<7 cm2	7/25 (14.6)	10/25 (40.0)	1.70 (0.64-4.48)	
≥8 cm2	71/134 (53.0)	50/133 (37.6)	0.64 (0.45-0.92)	0.101
<8 cm2	10/37 (27.0)	15/37 (40.5)	1.76 (0.79-3.92)	
≥9 cm2	68/124 (54.8)	46/123 (37.4)	0.62 (0.42-0.90)	0.011
<9 cm2	13/47 (27.7)	19/47 (40.4)	1.65 (0.81-3.35)	
≥10 cm2	65/117 (55.6)	45/118 (38.1)	0.62 (0.42-0.91)	0.022
<10 cm2	16/54 (29.6)	20/52 (38.5)	1.42 (0.73-2.74)	
≥11 cm2	57/104 (54.8)	40/106 (37.7)	0.63 (0.42-0.94)	0.064
<11 cm2	24/67 (35.8)	25/64 (39.1)	1.14 (0.65-2.00)	
≥12 cm2	54/97 (55.7)	38/98 (38.8)	0.64 (0.42-0.96)	0.097
<12 cm2	27/74 (36.5)	27/72 (37.5)	1.06 (0.62-1.81)	
≥13 cm2	49/89 (55.1)	32/87 (36.8)	0.60 (0.38-0.94)	0.076
<13 cm2	32/82 (39.0)	33/83 (39.8)	1.06 (0.65-1.72)	
≥14 cm2	48/87 (55.2)	27/77 (35.1)	0.56 (0.35-0.90)	0.043
<14 cm2	33/84 (39.3)	38/93 (40.9)	1.08 (0.68-1.72)	
≥15 cm2	45/81 (55.6)	25/72 (34.7)	0.54 (0.33-0.89)	0.044
<15 cm2	36/90 (40.0)	40/98 (40.8)	1.06 (0.68-1.66)	
≥16 cm2	43/76 (56.6)	24/70 (34.3)	0.52 (0.32-0.86)	0.031
<16 cm2	38/95 (40.0)	41/100 (41.0)	1.07 (0.69-1.66)	
≥17 cm2	39/66 (59.1)	20/63 (31.7)	0.45 (0.26-0.78)	0.008
<17 cm2	42/105 (40.0)	45/107 (42.1)	1.09 (0.72-1.67)	
≥18 cm2	37/63 (58.7)	20/61 (32.8)	0.48 (0.28-0.82)	0.017
<18 cm2	44/108 (40.7)	45/109 (41.3)	1.04 (0.69-1.58)	
≥19 cm2	36/61 (59.0)	18/53 (34)	0.51 (0.29-0.89)	0.032
<19 cm2	45/110 (40.9)	47/117 (40.2)	0.99 (0.66-1.50)	
≥20 cm2	34/59 (42.0)	17/49 (34.7)	0.54 (0.30-0.96)	0.074
<20 cm2	47/112 (58.0)	48/121 (39.7)	0.95 (0.63-1.42)	
≥21 cm2	33/57 (57.9)	16/47 (34.0)	0.52 (0.29-0.95)	0.064
<21 cm2	48/114 (42.1)	49/123 (39.8)	0.95 (0.64-1.41)	
≥22 cm2	33/55 (60.0)	15/42 (35.7)	0.53 (0.29-0.97)	0.065
<22 cm2	48/116 (41.4)	50/128 (39.1)	0.94 (0.64-1.40)	
≥23 cm2	28/47 (59.6)	15/41 (36.6)	0.53 (0.28-0.99)	0.121
<23 cm2	53/124 (42.7)	50/129 (38.8)	0.91 (0.62-1.34)	
≥24 cm2	25/43 (58.1)	15/41 (36.6)	0.55 (0.29-1.05)	0.183
<24 cm2	56/128 (43.7)	51/129 (38.8)	0.88 (0.60-1.29)	
≥25 cm2	21/38 (55.3)	13/38 (34.2)	0.54 (0.27-1.09)	0.234
<25 cm2	60/133 (45.1)	52/132 (39.4)	0.87 (0.60-1.26)	

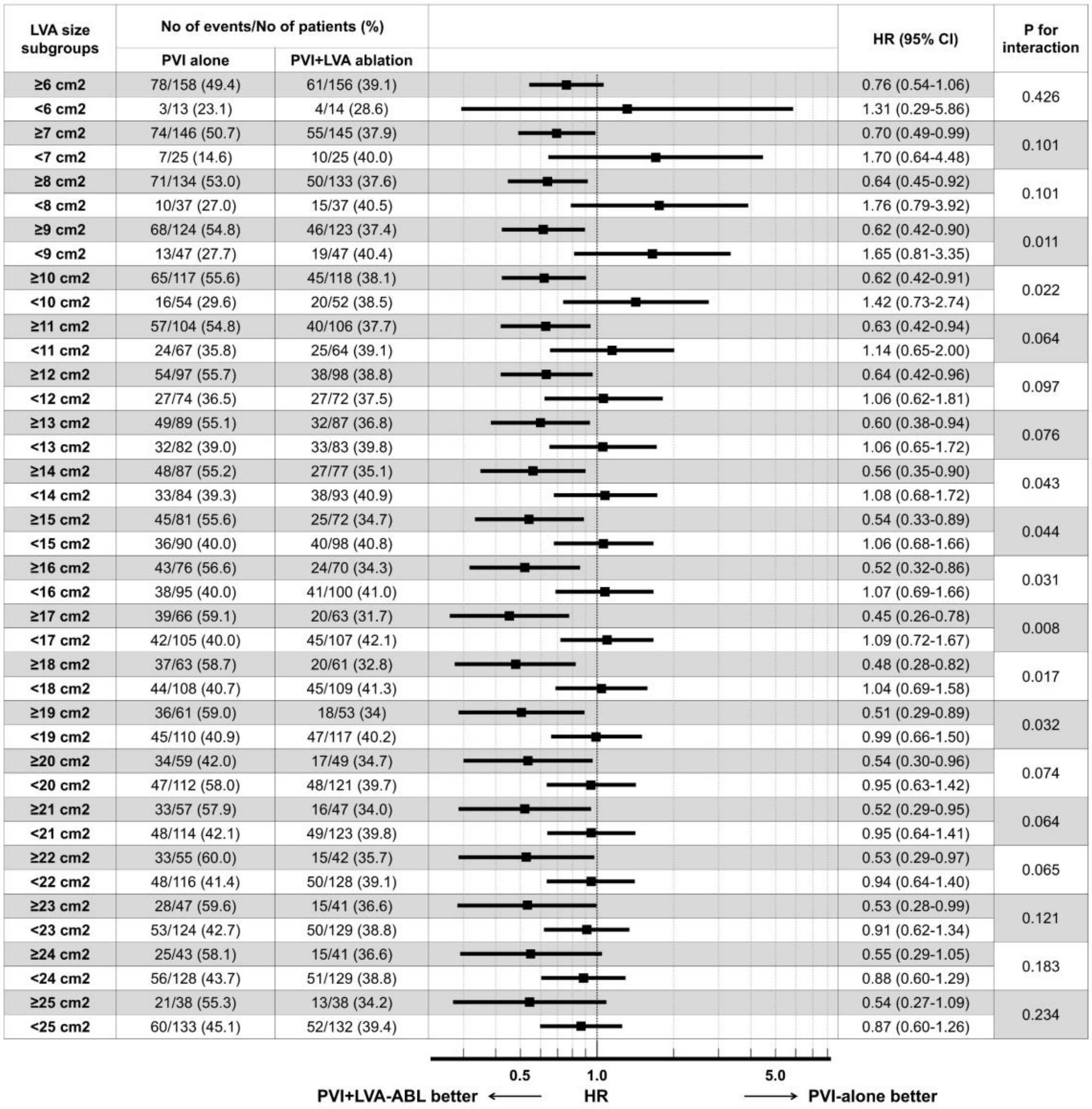

Forest plots showing HRs with 95% CIs for AF/AT recurrence comparing PVI+LVA-ABL with PVI-alone, stratified by LVA size. In 1-cm² increment analyses, adjunctive LVA ablation demonstrated consistent benefit from ≥7 cm², with the strongest treatment-by-size interaction observed at 17 cm² (p for interaction = 0.008).

AF, atrial fibrillation; AT, atrial tachycardia; CI, confidence interval; HR, hazard ratio; LVA, low-voltage area; PVI, pulmonary vein isolation; PVI+LVA-ABL, pulmonary vein isolation followed by low-voltage area ablation

Figure S5. Restricted cubic spline analyses of treatment effect across LVA size stratified by left atrial diameter

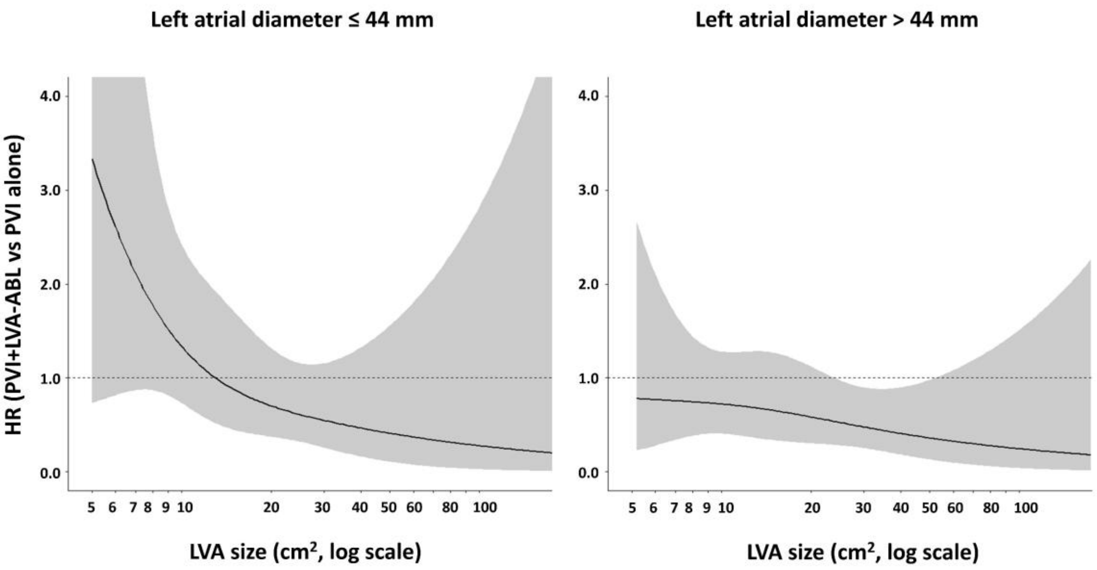

Restricted cubic spline Cox models showing the estimated hazard ratio for PVI plus LVA ablation versus PVI alone across LVA size, stratified by left atrial diameter (≤44 mm and >44 mm). Shaded areas indicate 95% confidence intervals. Axes are identical across panels. LVA size is shown on a log scale. There was no evidence that the continuous treatment-by–low-voltage area association differed by left atrial diameter strata (three-way interaction P = 0.556).

HR, hazard ratio; LVA, low-voltage area

Figure S6. Cumulative incidence of AF recurrence

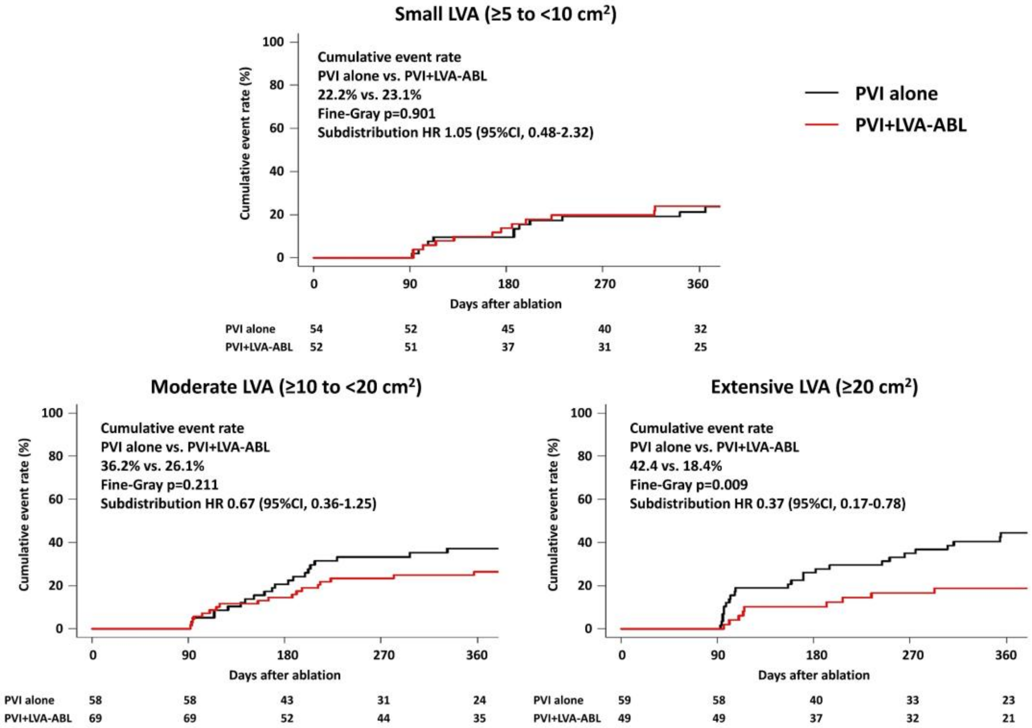

Cumulative incidence of AF recurrence, analyzed separately from AT recurrence. No significant differences were observed between PVI+LVA-ABL and PVI-alone groups in patients with small or moderate LVAs. In contrast, AF recurrence rates were significantly lower with PVI+LVA-ABL than with PVI alone in patients with extensive LVAs. AT recurrence (n = 35) and death (n = 3) were analyzed as competing events using the Fine–Gray method.

AF, atrial fibrillation; CI, confidence interval; HR, hazard ratio; LVA, low-voltage area; PVI, pulmonary vein isolation; PVI+LVA-ABL, pulmonary vein isolation followed by low-voltage area ablation

Figure S7. Cumulative incidence of AT recurrence

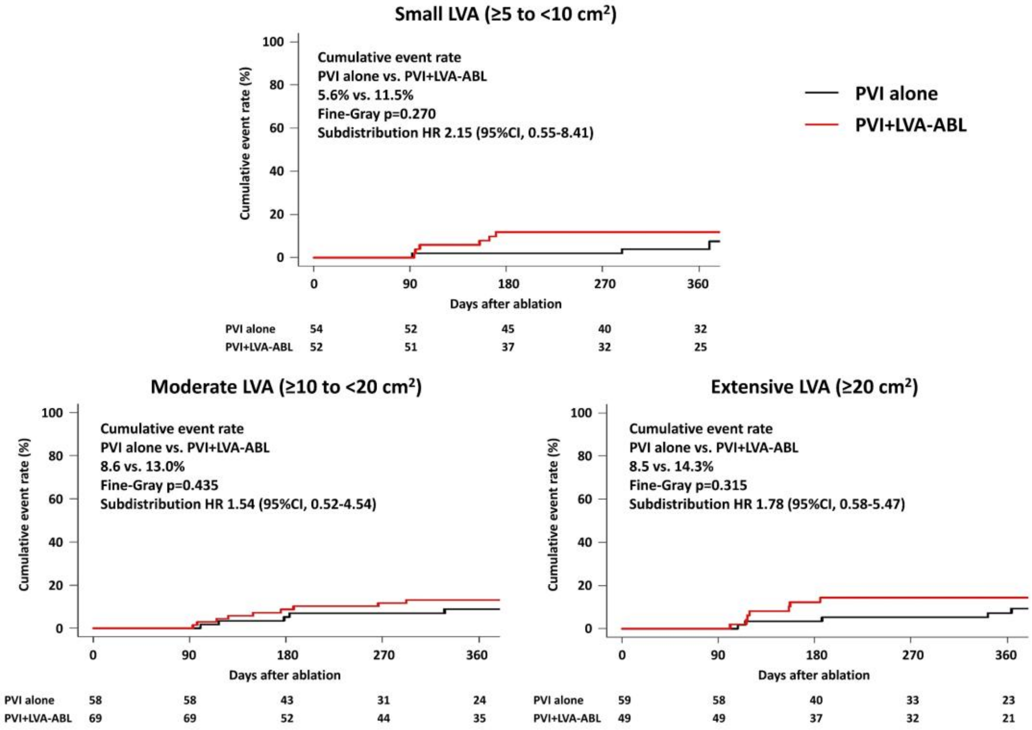

Cumulative incidence of AT recurrence, analyzed separately from AF recurrence. Although not statistically significant, AT recurrence rates were numerically higher with PVI+LVA-ABL than with PVI-alone across all LVA size categories. AF recurrence (n = 97) and death (n = 3) were analyzed as competing events using the Fine–Gray method.

AF, atrial fibrillation; AT, atrial tachycardia; LVA, low-voltage area; PVI, pulmonary vein isolation; PVI+LVA-ABL, pulmonary vein isolation followed by low-voltage area ablation
